# Association Between Recent Mechanical Risk Factors for Venous Thrombosis and Young-Onset Cryptogenic Ischemic Stroke

**DOI:** 10.1161/STROKEAHA.125.054299

**Published:** 2026-08-26

**Authors:** Julija Valančienė, Pauli Ylikotila, Riikka Lautamäki, Marialuisa Zedde, Teresa Grimaldi, Tomi Sarkanen, Essi Ryödi, Dalius Jatužis, Kristina Ryliškienė, Diana Zakarkaitė, Bettina von Sarnowski, Raila Busch, Lauri Tulkki, Jani Pirinen, Nicolas Martinez-Majander, Radim Licenik, Phillip Ferdinand, Cheryl Oxley, Janika Kõrv, Piibe Muda, Tuukka A. Helin, Lotta Joutsi-Korhonen, Tímea Szántó, Alessandro Pezzini, Carlo Mario Lombardi, Georgios Tsivgoulis, Satu Suihko, Heli Tolppanen, Ana Catarina Fonseca, Patricia Martínez-Sánchez, Laura Amaya-Pascasio, Sofie Abels, Frank-Erik de Leeuw, Nilufer Yesilot, Mine Sezgin, Ulrike Waje-Andreassen, Sahrai Saeed, Petra Redfors, Odd Bech-Hanssen, Juha Huhtakangas, Marja Hedman, Pekka Jäkälä, Hugo ten Cate, Eva Gerdts, Mika Lehto, Juha Sinisalo, Jukka Putaala, on behalf of the SECRETO Study Group Investigators, Külliki Karu

**Affiliations:** Department of Neurology, University of Helsinki and Helsinki University Hospital, Finland (J.V.).; Department of Neurology and Neurosurgery (J.V., D.J., K.R.), Faculty of Medicine, Institute of Clinical Medicine, Vilnius University, Lithuania.; Department of Cardiac and Vascular Diseases (D.Z.), Faculty of Medicine, Institute of Clinical Medicine, Vilnius University, Lithuania.; Department of Neurology, Turku University Hospital and University of Turku, Finland (P.Y.).; Heart Center, Turku University Hospital, Finland (R. Lautamäki).; Neurology Unit, Stroke Unit (M.Z.), Azienda Unità Sanitaria Locale-IRCCS Reggio Emilia, Italy.; Cardiology Unit (T.G.), Azienda Unità Sanitaria Locale-IRCCS Reggio Emilia, Italy.; Department of Neurology, Tampere University Hospital, Wellbeing Services County of Pirkanmaa and Faculty of Medicine and Health Technology, Tampere University, Finland (T. Sarkanen).; Heart Hospital, Tampere University Hospital, Finland (E.R.).; Department of Neurology (B.v.S.), University Medicine Greifswald, Germany.; Department of Internal Medicine B (Cardiology) (R.B.), University Medicine Greifswald, Germany.; Department of Neurology (L.T., N.M.-M., J. Putaala), Helsinki University Hospital and University of Helsinki, Finland.; Department of Cardiology, Heart and Lung Center (J. Pirinen, S. Suihko, H.T., M.L., J.S.), Helsinki University Hospital and University of Helsinki, Finland.; Department of Clinical Chemistry, HUS Diagnostic Center (T.A.H., L.J.-K.), Helsinki University Hospital and University of Helsinki, Finland.; Acute Stroke Centre, North West Anglia Foundation NHS Trust, Peterborough, United Kingdom (R. Licenik).; Neurosciences (P.F.), University Hospitals of North Midlands NHS Trust, Stoke-on-Trent, United Kingdom.; Cardiac Physiology Services (C.O.), University Hospitals of North Midlands NHS Trust, Stoke-on-Trent, United Kingdom.; Department of Neurology and Neurosurgery (J.K.), University of Tartu, Estonia.; Department of Cardiology (P.M.), University of Tartu, Estonia.; Unit of Coagulation Disorders, Department of Hematology, Helsinki University Hospital Comprehensive Cancer Center and University of Helsinki, Finland (T. Szántó).; Department of Medicine and Surgery, University of Parma, Italy (A.P.).; Cardiology, ASST Spedali Civili di Brescia and Department of Medical and Surgical Specialties, Radiological Sciences, and Public Health, University of Brescia, Italy (C.M.L.).; Second Department of Neurology, National & Kapodistrian University of Athens, Attikon University Hospital, Greece (G.T.).; Faculty of Medicine, Hospital Santa Maria, University of Lisbon, Portugal (A.C.F.).; Department of Neurology, Torrecardenas University Hospital, University of Almería, Spain (P.M.-S., L.A.-P.).; Department of Cardiology, Radboud University Medical Center, Nijmegen, the Netherlands (S.A.).; Department of Neurology, Radboud Institute of Medical Innovation, Donders Institute for Brain, Cognition and Behavior, Nijmegen, the Netherlands (F.-E.d.L.).; Department of Neurology (N.Y.), Istanbul University, Istanbul Faculty of Medicine, Turkey.; Department of Cardiology (M.S.), Istanbul University, Istanbul Faculty of Medicine, Turkey.; Department of Neurology (U.W.-A.), Haukeland University Hospital, Bergen, Norway.; Department of Heart Disease (S. Saeed), Haukeland University Hospital, Bergen, Norway.; Department of Neurology, Sahlgrenska University Hospital and Department of Clinical Neuroscience, Institute of Neuroscience and Physiology (P.R.), Sahlgrenska Academy at University of Gothenburg, Sweden.; Department of Clinical Physiology, Institute of Medicine (O.B.-H.), Sahlgrenska Academy at University of Gothenburg, Sweden.; Department of Neurology, Oulu University Hospital and University of Oulu, Finland (J.H.).; Heart Center, Kuopio University Hospital, Finland (M.H.).; Neurocenter Neurology, Kuopio University Hospital and University of Eastern Finland, Finland (P.J.).; Department of Internal Medicine and Thrombosis Expertise Center, Maastricht University Medical Center and CARIM School for Cardiovascular Diseases, the Netherlands (H.t.C.).; Department of Clinical Science, Center for Research on Cardiac Disease in Women, University of Bergen, Norway (E.G.).; Stroke Care Program, Department of Emergency, Parma University Hospital, Italy (A.P.).

**Keywords:** case-control studies, embolism, foramen ovale, ischemic stroke, patent, risk factors, thrombophilia

## Abstract

**BACKGROUND:**

In young adults, the risk factors (RFs) for cryptogenic ischemic stroke (CIS) remain unclear. We aimed to investigate the association between recent mechanical venous thrombosis RFs (VTRFs) and young-onset CIS in this multicenter sex- and age-matched case-control study.

**METHODS:**

Five hundred forty-six patients aged 18 to 49 years (median age, 41 [34–46] years; 47.3% women) with a recent CIS and 546 stroke-free community-based controls were included in the study across 19 European centers between October 2013 and November 2022. Mechanical VTRFs, including surgical treatment, nonsurgical intervention, injury, and immobilization occurring within 90 days prestroke, were assessed using standardized, structured interviews. Conditional logistic regression was used to assess the association of mechanical VTRFs with CIS. Age, sex, level of education, and traditional and nontraditional stroke RFs were included as confounders.

**RESULTS:**

Mechanical VTRFs within 14 days prestroke were more prevalent in cases than controls (15.4% versus 9.6%) and independently associated with CIS after adjustment for demographics and traditional stroke RFs (odds ratio [OR], 1.67 [95% CI, 1.05–2.67]). In age-stratified analysis, this association remained significant after additional adjustment for nontraditional RFs in 18- to 39-year-olds (OR, 2.36 [95% CI, 1.02–5.46]) but was not significant in the 40- to 49-year age group (OR, 1.50 [95% CI, 0.73–3.08]). In addition, mechanical VTRFs were associated with CIS in cases with clinically relevant patent foramen ovale in a fully adjusted model (OR, 2.06 [95% CI, 1.21–3.51]) but not in those without (OR, 1.29 [95% CI, 0.76–2.18]). No significant associations were observed for mechanical VTRFs occurring >14 days before stroke. Baseline blood thrombophilia markers did not differ between cases with and without mechanical VTRFs 0 to 14 or 15 to 90 days before stroke.

**CONCLUSIONS:**

Recent mechanical VTRFs occurring within 14 days preceding stroke contribute to the risk of young-onset CIS, particularly in younger individuals and those with a clinically relevant patent foramen ovale. This risk does not appear to be conveyed by thrombophilic factors assessed.

**REGISTRATION:**

URL: https://www.clinicaltrials.gov; Unique identifier: NCT01934725.

Despite the rising incidence of ischemic stroke among young individuals, a comprehensive understanding of the pathophysiological factors remains lacking. This is evidenced by the fact that ≈25% to 50% of patients have no identifiable cause of stroke at a young age, that is, cryptogenic ischemic stroke (CIS).^1–4^ While traditional cardiovascular risk factors (RFs) and their increasing prevalence are known to be associated with young-onset stroke, there remains a gap in understanding the role of nontraditional RFs and other potential, for example, temporal, contributors.^5^ However, emerging evidence suggests that these nontraditional factors may be equally or even more important, particularly in younger stroke populations.^6^

Patent foramen ovale (PFO), one of the specific phenotypic features considered in young stroke patients, is a prevalent cardiac anomaly occurring in ≈25% of the general population.^7^ Nevertheless, its direct role in the cause of ischemic stroke remains inconclusive, particularly given its high prevalence.^8^ The causal relationship between PFO and stroke is generally dependent on additional factors or conditions, such as the presence of a significant shunt, atrial septal aneurysm, or specific prothrombotic states. These factors may increase the risk of venous thrombosis (VT), which can subsequently result in paradoxical embolization from a venous source, causing a PFO-associated stroke.^8–10^

The established triggering RFs for VT include transient mechanical factors, such as surgery, injury, trauma, or immobilization.^11,12^ Emerging evidence suggests a potential link between these triggers and PFO-associated stroke. However, it remains unclear whether these factors are disproportionately prevalent in CIS cases with a PFO compared with those without.^8,13^ Moreover, the potential for mechanical VTRFs to provoke systemic hypercoagulability or interact with inherited thrombophilias in CIS remains unstudied.^6,14^ Finally, while the temporal relationship between exposure to triggering VTRFs and subsequent thrombotic events is generally estimated to be about 3 months, the precise timing remains poorly characterized.^12^

To our knowledge, no prior studies have specifically investigated the association between transient mechanical VTRFs and CIS in young adults. Thus, in this international multicenter case-control study, we aimed to explore the associations between any mechanical VTRFs occurring at distinct time intervals before stroke onset and CIS in young adults (aged 18–49 years), with stratified analyses by PFO status, sex, and age. In addition, we compared markers of hypercoagulability between patients with CIS with and without preceding VTRFs.

## Methods

The data supporting the findings of this study are not publicly available but are available upon reasonable request and with permission of the SECRETO consortium (Searching for Explanations for Cryptogenic Stroke in the Young: Revealing the Etiology, Triggers, and Outcome). This article follows the STROBE reporting guidelines (Strengthening the Reporting of Observational Studies in Epidemiology; Supplemental Material).

### Study Population

We obtained data from the prospective international multicenter case-control study SECRETO (Unique identifier: NCT01934725). The study enrolled patients aged 18 to 49 years who had a first-ever radiologically proven CIS and were hospitalized and investigated using a standardized protocol in 19 European centers in 13 countries (detailed in Figure S1).^15^ Participants were enrolled between October 2013 and November 2022. Each patient with CIS (case) was matched 1:1 with a stroke-free control of the same sex, age (±5 years), and ethnicity. Controls were primarily recruited from population-based sources or, when unavailable, from nonrelated proxies. To minimize residential and socioeconomic confounding, all controls were recruited from the same geographic region (eg, hospital catchment area). Throughout this study, the term participants is used to refer to the total population of both cases and controls.

CIS was defined to be present if the 4 phenotypes according to the Atherosclerosis, Small Vessel Disease, Cardiac Source, Other Cause (A-S-C-O) classification system were graded as 0 (no disease), grade 2 (causality uncertain), or grade 3 (unlikely to represent a direct cause of the index stroke).^16^ However, due to the uncertain role in the cause of stroke, patients with PFO or individual coagulopathies, except for established antiphospholipid syndrome, were also included in the study.

All enrolled patients (cases) had to undergo a protocol workup to rule out definitive causes of stroke. This included magnetic resonance imaging of the brain, imaging of the intracranial and extracranial vessels with either computed tomography angiography or magnetic resonance angiography, routine laboratory tests, 12-lead ECG and continuous ECG for at least 24 hours, and both transthoracic echocardiography and transesophageal echocardiography and laboratory screening for the most common thrombophilias. Stroke severity was assessed by the National Institutes of Health Stroke Scale score.

The study was approved by the local ethics committees at each recruiting center. Signed written informed consent was obtained from each participant or their legal representative.

### Chronic Stroke RFs and Comorbidities

Information on demographics, socioeconomic status, some comorbidities, and lifestyle RFs was obtained through standardized, structured in-person interviews conducted during the study visit by trained personnel blinded to study hypotheses at the participating centers at the time of recruitment for cases and controls. Socioeconomic status was assessed by the level of education, which was categorized as high (postsecondary nontertiary education or higher) or low (primary or lower/upper secondary). Ethnicity was categorized as White (European, Middle Eastern, and North African) or other. Migraine with aura was diagnosed using a validated migraine screening protocol,^17^ and depression was defined as experiencing low mood, sadness, or depression for at least 2 consecutive weeks in the past year.^18^ Women were asked if they were not currently pregnant or in puerperium (time period of 90 days from the end of pregnancy), had recurrent miscarriages or a history of perinatal conditions, including gestational diabetes, hypertension, preeclampsia, eclampsia, or hemolysis, elevated liver enzymes, and low platelets syndrome, or were using estrogen-containing contraceptives by any route of administration before the stroke. Participants were defined as physically inactive if the metabolic equivalents per week were <1500 using the self-administered format of the International Physical Activity Questionnaire.^19^ A modified version of the Mediterranean Diet Score was used to assess the diet of the participants, with a score of <25 being defined as unhealthy.^20^ Heavy alcohol use was defined as drinking alcohol on average at least twice a month and at least 5 (women) or 7 units (men) per occasion or >7 (women) or >14 units (men) per week based on the adapted version of the World Health Organization Alcohol, Smoking and Substance Involvement Screening Test.^21^ Current tobacco smoking was defined as smoking at least 1 cigarette a day on average. In addition, participants were also asked about having used illicit drugs in the previous 12 months using the Alcohol, Smoking and Substance Involvement Screening Test Questionnaire.^21^ Psychosocial stress was measured as an overall score combining work-related and domestic stress, and was categorized as either permanent stress or stress experienced in several periods, or no or occasional stress over the previous year.

Information regarding other chronic comorbidities and previous medical history was confirmed through systematic abstraction of medical records or direct clinical assessment of cases and controls during the baseline study visit. The assessed comorbidities included hypertension (previous diagnosis of hypertension, use of antihypertensive medication at the time of stroke, or mean office blood pressure ≥140/90 mm Hg on 2 assessments at baseline study visit), hypercholesterolemia, diabetes (previous diagnosis or the use of corresponding medication at the time of the stroke), obesity (waist-to-hip ratio >0.90 for men and 0.85 for women), cardiovascular disease (including any of the following: coronary heart disease, peripheral arterial disease, heart failure, arterial thrombosis, aneurysm, and aortic or valvular disease), history of obstructive sleep apnea, malignancy or VT (including deep vein thrombosis, pulmonary embolism, cerebral VT, unspecified VT), and any of the chronic multiorgan disorder, such as inflammatory bowel disease, other autoimmune disease, chronic kidney or liver disease, hematologic disease, or thrombophilia.

PFO was diagnosed with contrast-enhanced transcranial Doppler or transthoracic echocardiography/transesophageal echocardiography. For the present analysis, clinically relevant PFO was defined as the presence of a moderate to severe shunt or atrial septal defect/atrial septal aneurysm.^22,23^ In addition, a subset of stroke-free controls was systematically assessed for PFO at selected study centers depending on ethics approvals and site preferences (Table S1).

### Mechanical RFs for VT

Mechanical VTRFs within 3 months preceding stroke (for cases) or the study visit (for controls) were inquired for all study participants with a structured questionnaire and categorized as major surgical procedure (including dental procedures, but excluding minor procedures, such as skin incision and suturing, wound suturing, or tooth filling), major nonsurgical procedure or diagnostic test (including biopsy, catheterization, and oral examination by a dentist or dental hygienist), major injury (eg, muscle tear or sprain and fracture), and immobilization (eg, plaster cast, bed rest at home for at least 4 days, hospitalization, and long-distance travel lasting >4 hours). In the absence of a standardized system, we categorized mechanical VTRFs based on expert consensus. We included a broad spectrum of exposures acknowledging that even minor exposures may increase the risk of thrombosis, especially when combined with other prothrombotic factors.^11,12,24^

### Baseline Coagulation Markers

Levels of coagulation proteins, free protein S antigen, activities of antithrombin III, factor VIII, VWF (von Willebrand Factor), and fibrinogen concentration, were measured in cases at the time of recruitment (baseline visit) and at the 3-month visit, while these were measured at a single time point for stroke-free controls. We processed the samples using a standardized protocol and transferred them for storage at −80 °C to the Biobank Core Lab at the Institute for Molecular Medicine Finland, University of Helsinki. Samples were centrally analyzed at HUS Diagnostic Center, Clinical Chemistry Laboratory, Helsinki, Finland, using standardized and accredited protocols. In addition, cases underwent a standardized thrombophilia screen upon admission according to local laboratory protocols. This included testing for anticardiolipin antibodies, lupus anticoagulant, anti-β2-glycoprotein-I antibodies, factor V Leiden (G1691A) and prothrombin (G20210A) mutations, homocysteine levels, and functional assays for antithrombin III, protein C, and protein S. Standard coagulation tests (prothrombin time and thrombin time) were also performed.

### Statistical Analysis

Statistical analysis was performed using IBM SPSS Statistics 29.0 and R (R Core Team 2023). We compared baseline demographic characteristics and stroke RFs between cases with CIS and stroke-free controls using the Pearson χ^2^ test, the Fisher exact test, or the Wilcoxon rank-sum test, presenting results for the overall population and stratified by sex. The findings are presented as absolute numbers with percentages for categorical variables and as medians with interquartile ranges for continuous variables. Baseline coagulation markers were compared between VTRF-exposed and VTRF-unexposed stroke cases, stratified by exposure timing. Univariable analyses were performed utilizing the McNemar test. The frequency of missing values is reported in the respective tables; analyses were performed on a complete-case basis. A *P* value below 0.05 was considered to indicate statistical significance.

To account for the matched design, conditional logistic regression was used to evaluate the association between any mechanical VTRFs (categorized by prestroke timing) and CIS. Based on previous data showing that majority of the VT events appear in the first 2 weeks after exposure to these RFs and our preliminary analyses demonstrating nonsignificant associations for intervals beyond 14 (15–30, 31–60, and 61–90) days, we focused our primary analysis on 2 clinically relevant periods: the acute (≤14 days) and subacute (15–90 days) prestroke intervals.^25^ Several models were used in the analysis: an unadjusted model, a model adjusted for age, sex, and level of education, and models accounting for traditional and nontraditional stroke RFs. The traditional stroke RFs included in the analysis were abdominal obesity, current tobacco smoking, depression, heavy alcohol consumption, hypertension, physical inactivity, psychosocial stress, and unhealthy diet. Factors such as diabetes, hypercholesterolemia, cardiovascular disease, and obstructive sleep apnea were excluded from the models due to their low prevalence. The nontraditional stroke RFs included chronic multiorgan disorder, a history of venous thromboembolism, illicit drug use, malignancy, and migraine with aura. These models were applied to the entire study population, age-stratified subgroups (18–39 versus 40–49 years), and sex-specific subgroups (female versus male). Formal tests for interaction between VTRFs and the matching variables (age and sex) were performed using product terms in fully adjusted unconditional logistic regression models. In the female subgroup, the conditional logistic regression model additionally incorporated female-specific RFs such as any perinatal condition or any prestroke estrogen preparation use. Due to low frequency, pregnancy or postpartum status was excluded as a covariate from the models. The adjusted odds ratios (ORs) and 95% CIs were reported across the models. In addition, a sensitivity analysis was performed by excluding major nonsurgical procedures or diagnostic tests from the mechanical VTRFs category, based on the presumption that these exposures may be associated with a lower prothrombotic risk.

To assess whether the association of mechanical VTRF is modified by the presence of a clinically relevant PFO, we performed binary logistic regression analyses stratified by cases’ PFO status. Cases were categorized by the presence of a clinically relevant PFO and compared with controls as a single reference group. In exploratory interaction analyses, we investigated potential interactions between mechanical VTRF timing and key variables, including age, sex, clinically relevant PFO, and burden of both traditional and nontraditional stroke RFs. We defined low burden of traditional stroke RFs as the presence of ≤2 factors and low burden of nontraditional stroke RFs as ≤1 factor (excluding clinically relevant PFO).^6^ In this analysis, we incorporated an interaction term into logistic regression models and calculated unadjusted ORs with CIs for each RF or subgroup in relation to any mechanical VTRFs occurring within 14 days preceding stroke. Certain RFs, including history of venous thromboembolism and clinically relevant PFO, were excluded from subgroup analyses due to insufficient case numbers for meaningful statistical comparison. In addition, to further investigate the origins of the potential interactions between age and mechanical VTRFs timing, an explicit descriptive subanalysis of raw exposure frequencies stratified by age groups was also performed.

## Results

Of 1165 individuals initially screened (608 cases and 557 potential controls), a total of 546 cases aged 18 to 49 years (median age, 41 [34–46] years; 47.25% women) with a recent CIS and 546 sex- and age-matched stroke-free controls (median age, 41 [34–46] years; 47.25% women) were included in the analysis (Figure S1).

### Univariate Comparison Between Cases and Matched Controls

CIS cases demonstrated significantly different demographic and clinical profiles compared with controls (Table 1). Cases had a lower educational level and showed a higher prevalence of traditional stroke RFs, including obesity, smoking, physical inactivity, heavy alcohol consumption, hypertension, depression, psychosocial stress, and unhealthy dietary habits. Hypercholesterolemia showed higher frequency in controls, albeit with low absolute prevalence. Nontraditional stroke RFs, namely, chronic multiorgan disorder, clinically relevant PFO, prior venous thromboembolism, and migraine with aura, were also more common among cases. Moreover, sex-specific differences were observed: only women in the patient group had higher rates of diabetes, hypertension, and prior venous thromboembolism, whereas only men in the patient group exhibited higher prevalence of depression, heavy alcohol consumption, and psychosocial stress alongside lower frequencies of hypercholesterolemia compared with the control group.

**Table 1.**
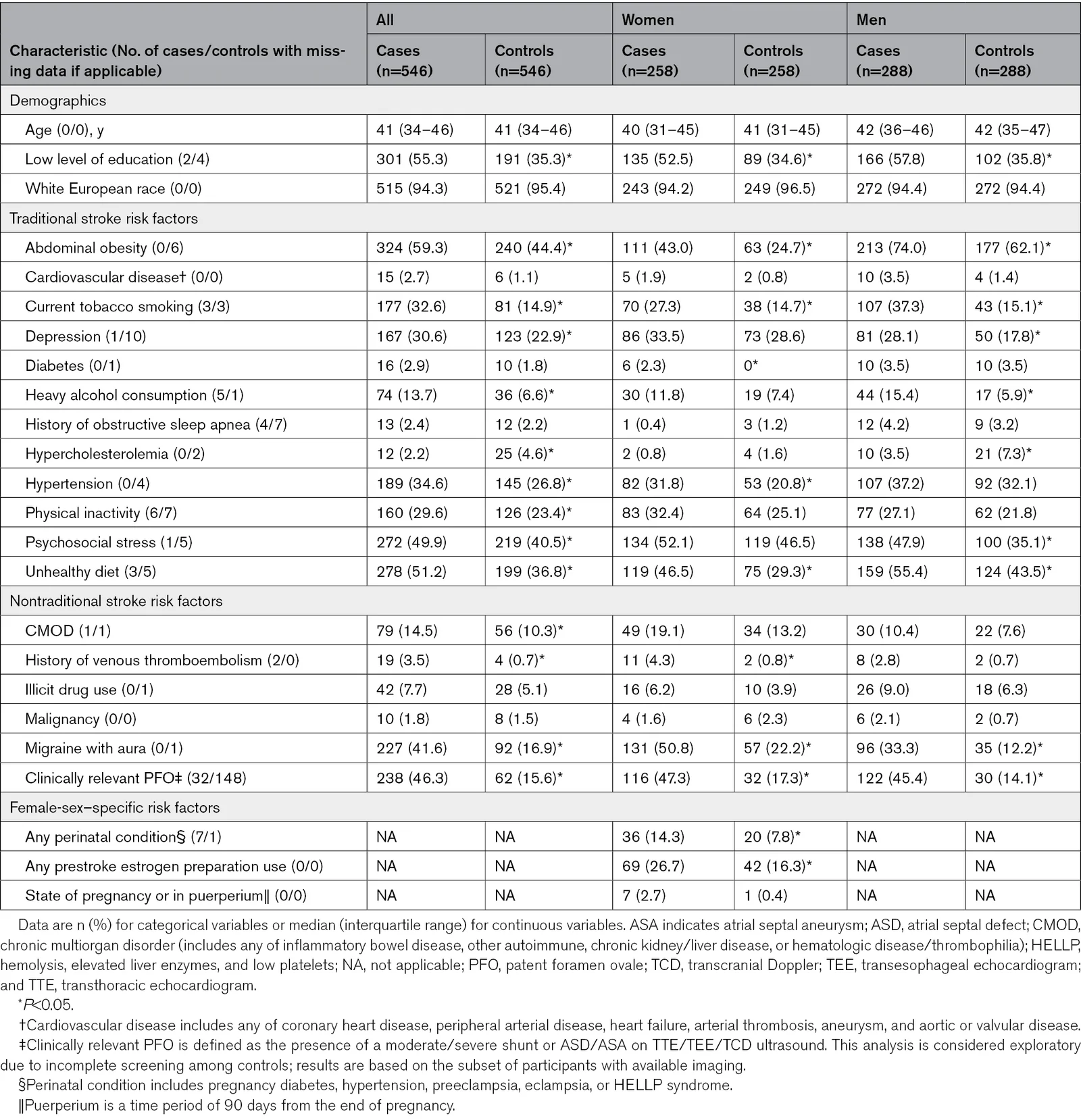
Comparison of Demographic Characteristics and Stroke Risk Factors of Young Cryptogenic Ischemic Stroke Cases and Stroke-Free Control Subjects Included in the Study

Any mechanical VTRFs occurring ≤14 days prestroke showed significantly higher prevalence in CIS cases versus controls in both the overall study population (15.4% versus 9.6%; *P*=0.004) and the male subgroup (16.7% versus 9.9%; *P*=0.031; Table 2). There were no differences in RFs occurring >14 days before stroke between groups. Furthermore, the co-occurrence of ≥2 VTRFs did not significantly differ between cases and controls at any time interval.

**Table 2.**
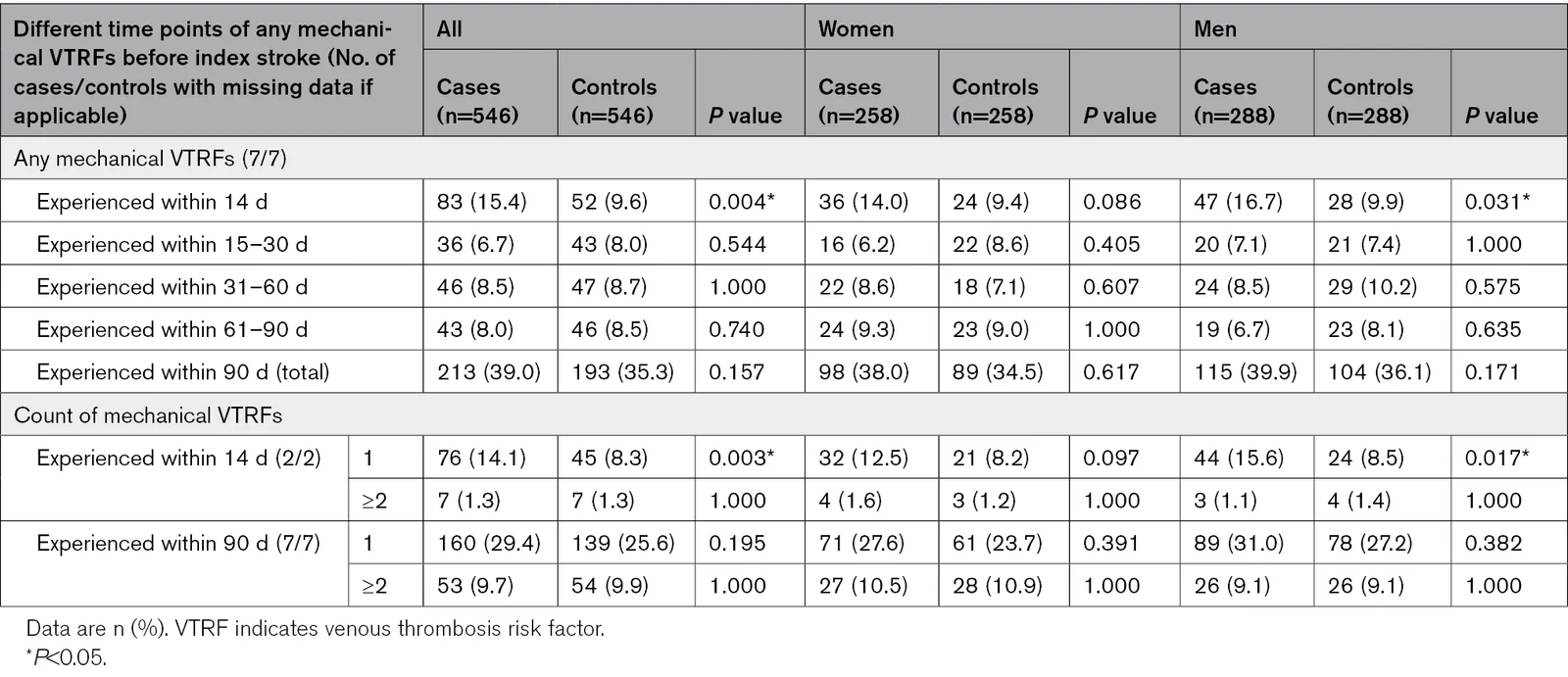
Comparison of Any Mechanical VTRFs at Different Time Points Before the Index Stroke Between Young Cryptogenic Ischemic Stroke Cases and Stroke-Free Controls

Subgroup analyses revealed distinct patterns among mechanical VTRFs (Figure 1). Recent immobilization (≤14 days prestroke) was significantly more prevalent in CIS cases than controls overall (10.1% versus 6.3%; *P*=0.021) and in men (12.9% versus 7.0%; *P*=0.018; Table S2). Injury showed an increased frequency ≤14 days before stroke in all cases (2.2% versus 0.7%; *P*=0.043) and for 31 to 60 days overall (2.8% versus 0.5%; *P*=0.004) and in female cases (2.7% versus 0.4%; *P*=0.033; Table S3). In contrast, neither surgical procedures nor nonsurgical interventions differed significantly between groups overall although surgical treatment within 14 days was more common in female cases than controls (4.7% versus 0.4%; *P*=0.002; Tables S4 and S5).

**Figure 1.**
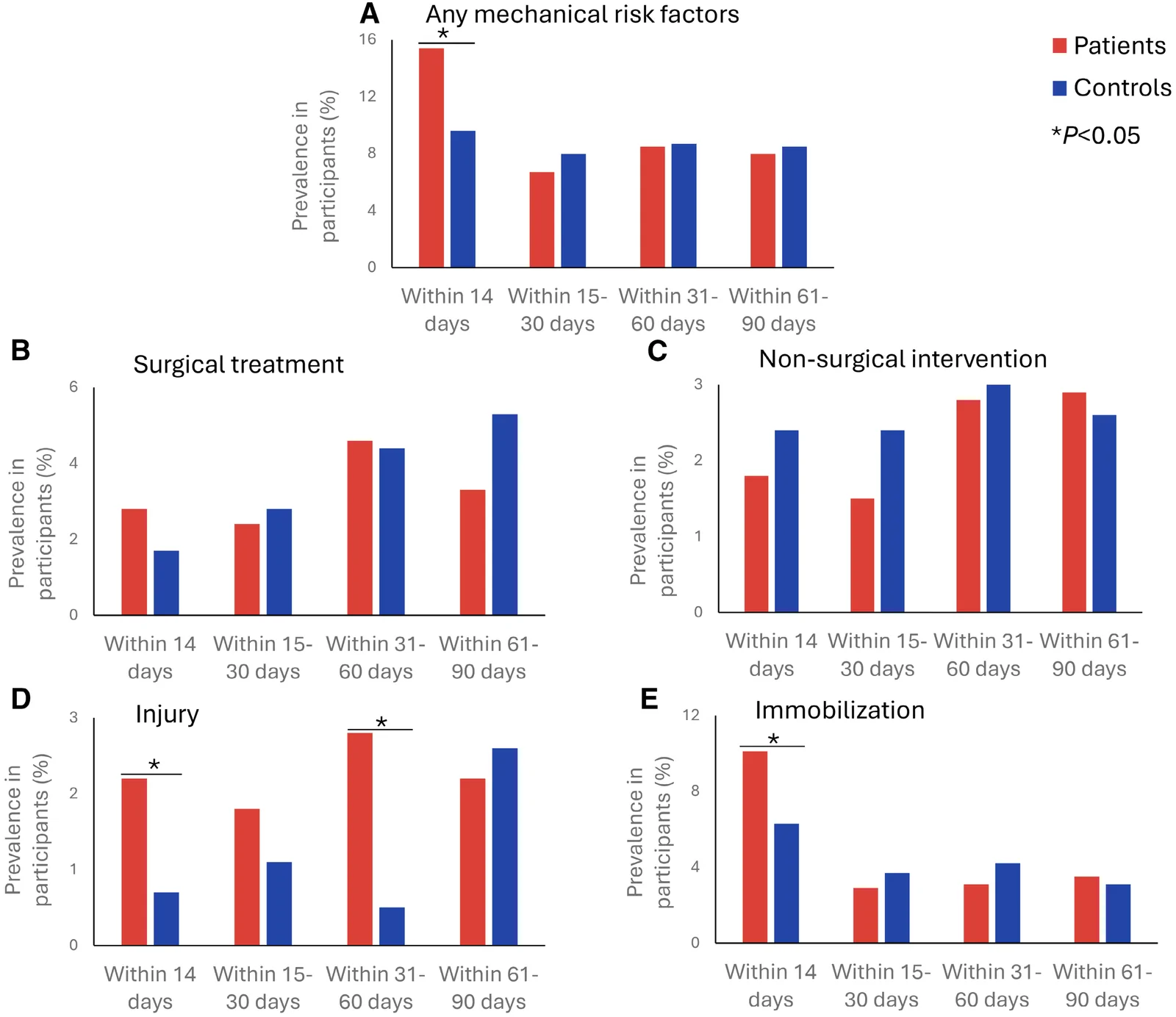
**Comparison of different mechanical risk factors for venous thrombosis at different time periods before the index stroke between young cryptogenic ischemic stroke cases and stroke-free controls. A**, Any mechanical risk factors. **B**, Surgical treatment. **C**, Nonsurgical intervention. **D**, Injury. **E**, Immobilization.

In age-stratified analysis, any mechanical VTRFs ≤14 days prestroke were more prevalent in cases than controls only in the 18- to 39-year age group (16.1% versus 8.5%; *P*=0.011). Within this subgroup, injury was the only category to reach statistical significance in this time window (3.6% versus 0.4%; *P*=0.021; Table S6).

### Association Between Mechanical VTRFs and CIS

Any mechanical VTRFs experienced within 14 days before stroke were significantly associated with CIS in the entire sample (Figure 2). This correlation was evident in the unadjusted model (OR, 1.78 [95% CI, 1.19–2.66]; *P*=0.005), the model adjusted for demographic variables (OR, 1.70 [95% CI, 1.11–2.59]; *P*=0.014), and the model further adjusted for traditional stroke RFs (OR, 1.67 [95% CI, 1.05–2.67]; *P*=0.032). In the model additionally adjusted for nontraditional stroke RFs, the association was close to significant (OR, 1.66 [95% CI, 1.00–2.77]; *P*=0.051; Table 3). In a sensitivity analysis excluding nonsurgical interventions from the VTRF definition, the association remained statistically significant across all models and demonstrated even higher effect size in adjusted models than in the primary analysis (Figure 3; Table S7).

**Table 3.**
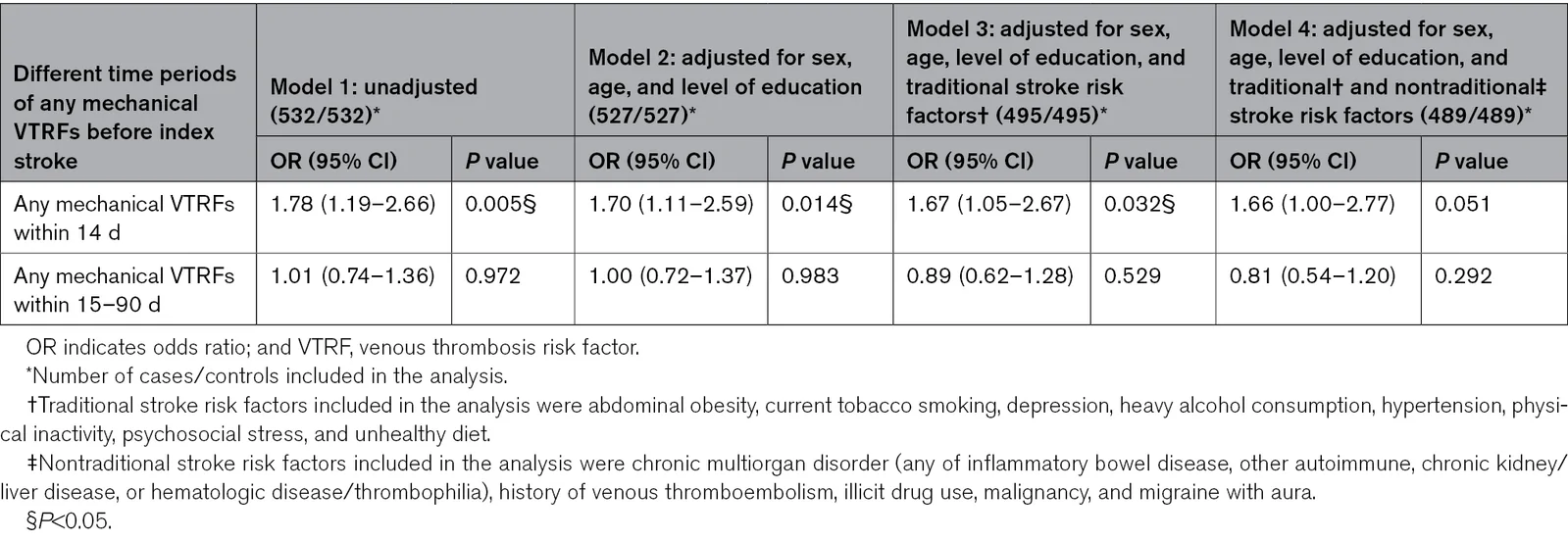
ORs and 95% CIs From Conditional Logistic Regression on the Association Between Any Mechanical VTRFs in Different Time Periods and Cryptogenic Ischemic Stroke in the Entire Sample

**Figure 2.**
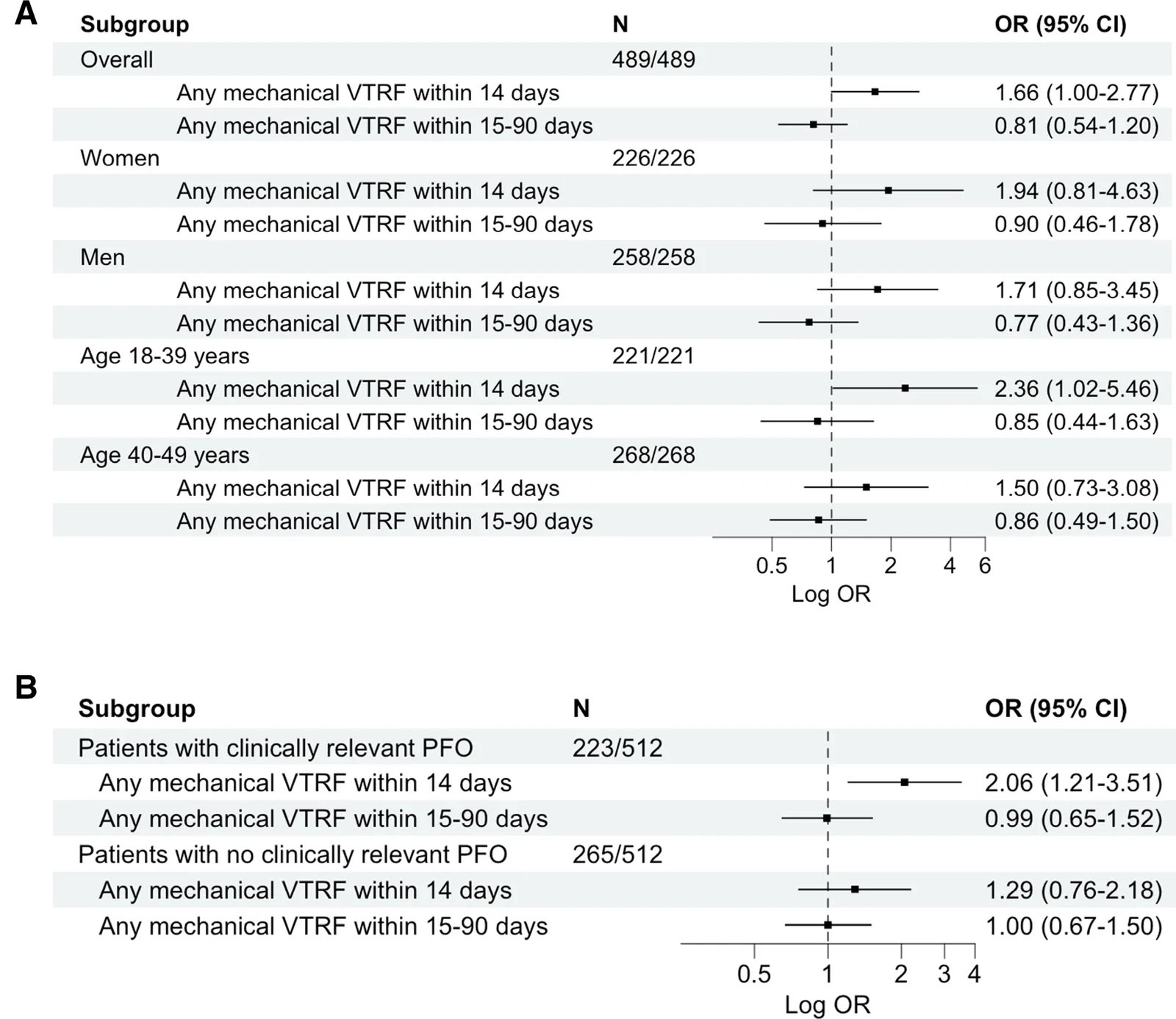
**Association between any mechanical venous thrombosis risk factors (VTRFs) at different time periods and cryptogenic ischemic stroke (CIS). A**, Odds ratios (ORs; 95% CIs) from conditional logistic regression analyses examining the association between any mechanical VTRFs timing and CIS: entire sample and sex-/age-stratified subgroups. The fully adjusted model included sex, age, level of education, traditional and nontraditional stroke risk factors, and female-sex–specific risk factors. **B**, ORs (95% CIs) from binary logistic regression analyses examining associations between any mechanical VTRFs timing and CIS. Cases were stratified by patent foramen ovale (PFO) status (present/absent), while controls were analyzed without PFO stratification due to incomplete data availability. The fully adjusted model included sex, age, level of education, and traditional and nontraditional stroke risk factors. Note: for all subgroups, N denotes the sample size formatted as (number of cases analyzed)/(number of controls analyzed).

**Figure 3.**
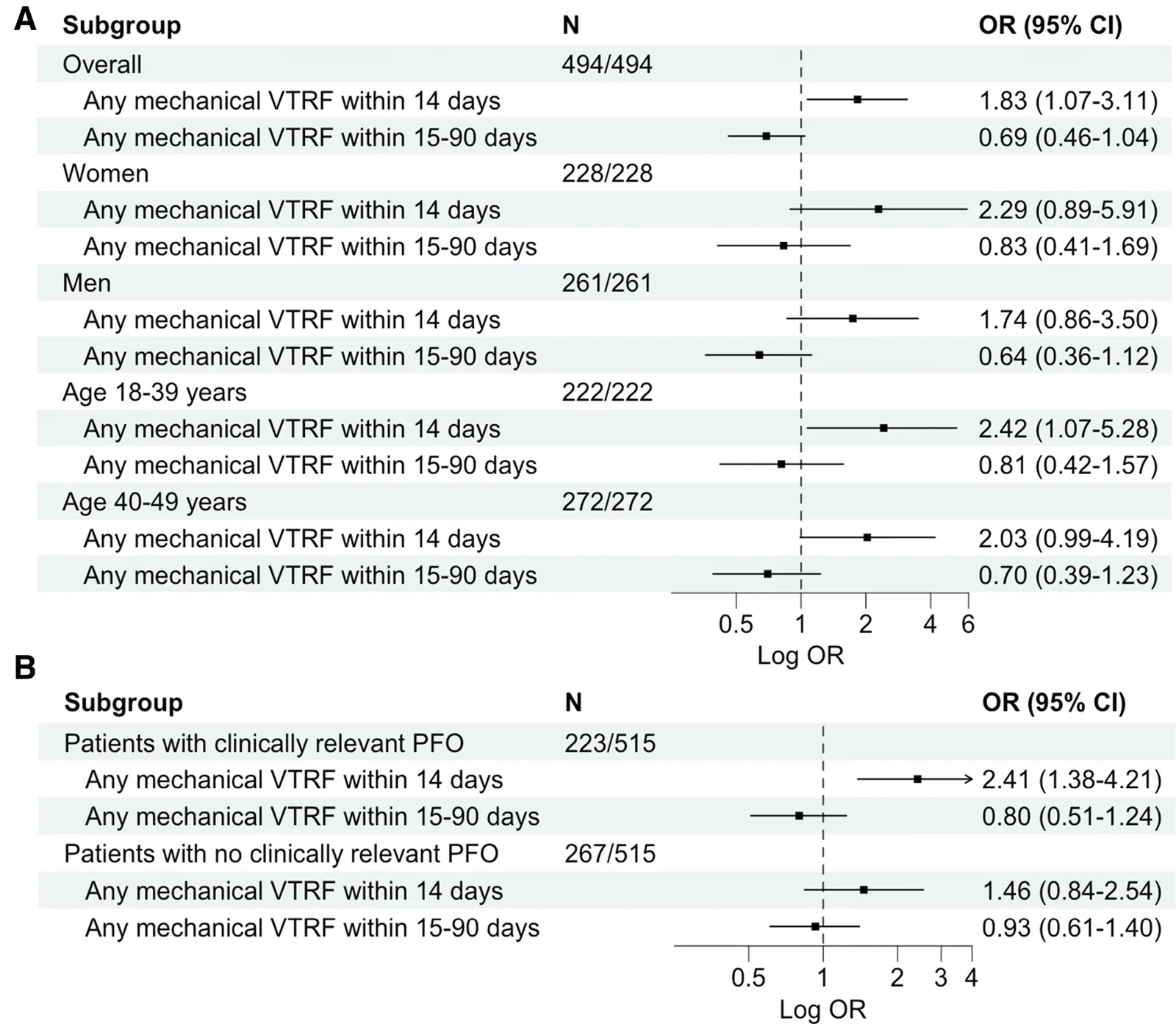
**Association between any mechanical venous thrombosis risk factors (VTRFs) in a sensitivity analysis excluding the category of nonsurgical interventions at different time periods and cryptogenic ischemic stroke (CIS). A**, Odds ratios (ORs; 95% CIs) from conditional logistic regression analyses examining the association between any mechanical VTRF (excluding nonsurgical interventions) timing and CIS: entire sample and sex-/age-stratified subgroups. The fully adjusted model included sex, age, level of education, traditional and nontraditional stroke risk factors, and female-sex–specific risk factors. **B**, ORs (95% CIs) from binary logistic regression analyses examining associations between any mechanical VTRFs (excluding nonsurgical interventions) timing and CIS. Cases were stratified by patent foramen ovale (PFO) status (present/absent), while controls were analyzed without PFO stratification due to incomplete data availability. The fully adjusted model included sex, age, level of education, and traditional and nontraditional stroke risk factors. Note: for all subgroups, N denotes the sample size formatted as (number of cases analyzed)/(number of controls analyzed).

Subgroup analyses stratified by sex revealed that the presence of any mechanical VTRF within the 14-day prestroke period was significantly associated with CIS only in the unadjusted model for men (OR, 1.77 [95% CI, 1.04–3.04]; *P*=0.037; Table S8). No statistical significance was observed in women across any models, including those adjusted for female-specific RFs (Table S9). Nevertheless, both subgroups exhibited a consistent trend of elevated risk (ORs >1) for CIS when mechanical VTRFs were present during the 14-day period across all models.

In age-stratified analyses, the presence of any mechanical VTRFs within 14 days was significantly associated with CIS in cases aged 18 to 39 years (adjusted OR, 2.36 [95% CI, 1.02–5.46]; *P*=0.046) after adjustment for age, education level, and traditional and nontraditional stroke RFs (Table S10). No significant association was found in cases aged 40 to 49 years across all models (fully adjusted OR, 1.50 [95% CI, 0.73–3.08]).

Notably, any mechanical VTRF occurring 15 to 90 days before stroke showed no association with CIS in the overall study population in either unadjusted or adjusted models. This null association persisted across all subgroup analyses (Figure 2), as well as in the sensitivity analysis (Table S7).

### Multivariable Analysis Stratified by PFO Status

Binary logistic regression analyses, stratified by PFO status, revealed that the presence of any mechanical VTRFs within 14 days was significantly associated with CIS exclusively among cases with clinically relevant PFO across all the models (fully adjusted OR, 2.06 [95% CI, 1.21–3.51]; *P*=0.008; Table 4). No significant association was observed in PFO-negative cases in any of the models. However, in a sensitivity analysis that excluded nonsurgical interventions, a significant association emerged in PFO-negative cases who had experienced any mechanical VTRFs within 14 days in unadjusted model (OR, 1.70 [95% CI, 1.06–2.74]; *P*=0.029) and persisted after adjustment for sex, age, and education level (OR, 1.79 [95% CI, 1.09–2.95]; *P*=0.023).

**Table 4.**
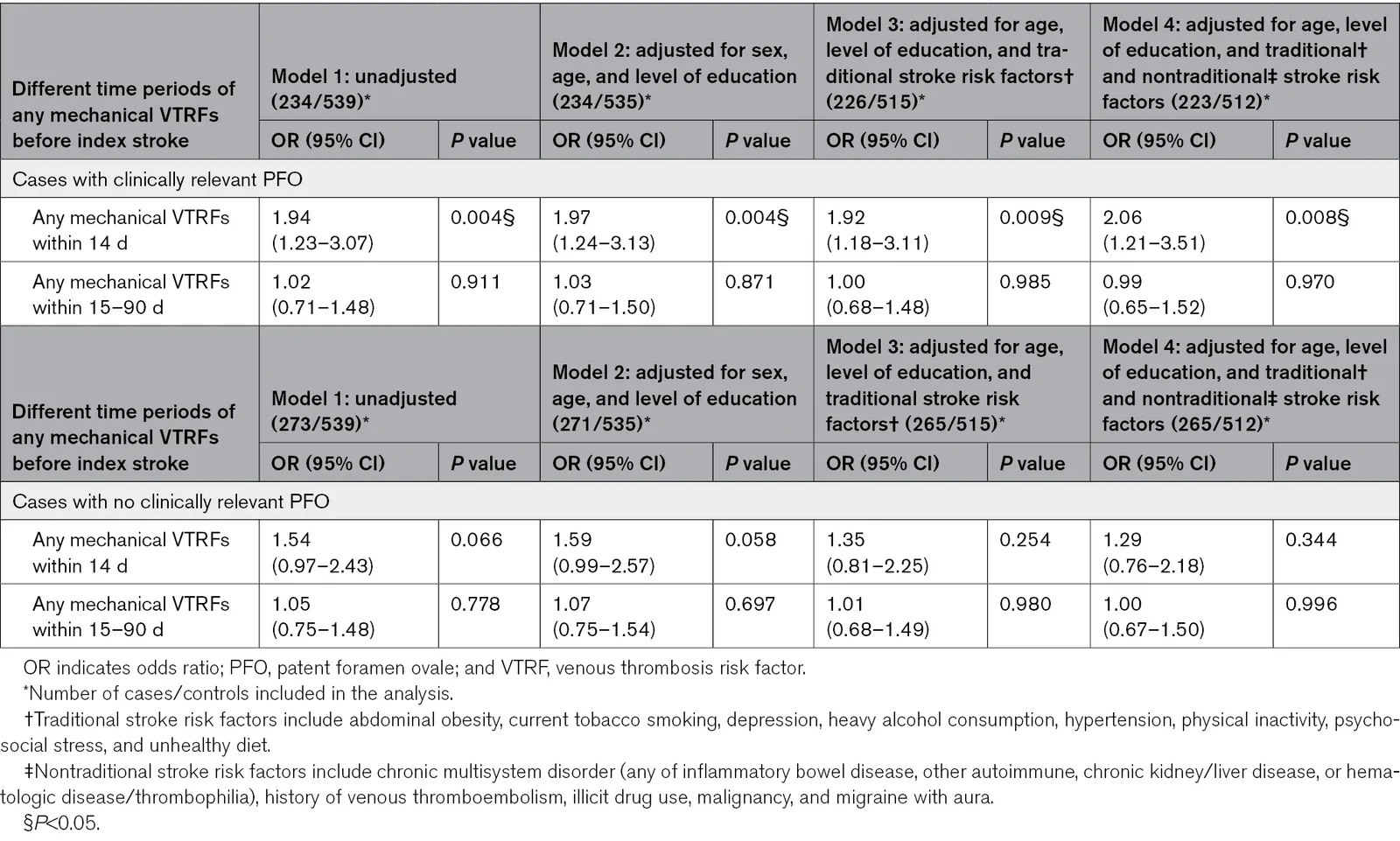
ORs and 95% CIs From Binary Logistic Regression on the Association Between Any Mechanical VTRFs in Different Time Points and Cryptogenic Ischemic Stroke With or Without Clinically Relevant PFO

### Interaction Assessment

In analysis of the total sample, we found no formal interaction between mechanical VTRFs and age (*P*=0.254), sex (*P*=0.993), clinically relevant PFO (*P*=0.322), or low burden of nontraditional stroke RFs (*P*=0.672; Table S11). However, a significant interaction emerged between mechanical VTRFs and low burden of traditional stroke RFs (*P*=0.044), with recent mechanical VTRFs (≤14 days prestroke) showing strong association in this subgroup (unadjusted OR, 2.97 [95% CI, 1.67–5.26]).

### Coagulation Markers in CIS Cases

The median time from stroke onset to laboratory testing was 7 (interquartile range, 4–11) days. Overall, baseline levels of coagulation proteins, including free protein S antigen, activities of antithrombin III, factor VIII, VWF, and fibrinogen concentration, did not differ significantly between patient groups: those without mechanical VTRFs, those with VTRFs within 14 days, and those within 15 to 90 days preceding stroke (Figure S2). Regarding routine thrombophilia test results, the prevalence of factor II G20210A mutation, factor V G1691A mutation, and the presence of any antiphospholipid antibodies did not differ between VTRF patient groups (Table S12). In an exploratory subgroup analysis, significant differences emerged in specific populations: VWF activity varied in cases aged 40 to 49 years; free protein S antigen levels, protein C, and antithrombin activity differed in cases with a high burden of traditional stroke RFs; and free protein S antigen levels differed in cases without clinically relevant PFO (Figures S2 through S8). A comparison of delta values (from baseline to 3 months) of coagulation marker levels between these VTRF groups showed a statistically significant difference only for VWF activity.

## Discussion

In this international multicenter case-control study, a significant independent association was identified between mechanical VTRFs within 14 days before stroke and CIS in young individuals after adjustment for demographic variables and traditional stroke RFs. This association holds true especially for younger individuals, those with a low burden of traditional stroke RFs, and those with a clinically relevant PFO. In a sensitivity analysis excluding nonsurgical interventions, this association remained statistically significant even after further adjustment for nontraditional stroke RFs. This suggests that not all VTRFs may confer equivalent risk, indicating that more invasive procedures play a greater role in triggering thrombotic events, a finding consistent with previous observations.^26^ Interestingly, we did not observe that mechanical VTRFs interacted with conventional thrombophilia factors, suggesting that in selected otherwise low-risk individuals, mechanical VTRF may act as a sole trigger factor for CIS. To the best of the authors’ knowledge, this study is the first to analyze the association of VTRFs, focusing on the entire young-onset CIS population, rather than solely on those with PFO.

While mechanical VTRFs are well-established contributors to VT through Virchow triad mechanisms (stasis, hypercoagulability, and endothelial injury) in low-flow venous systems, their role in arterial thrombosis remains understudied.^24^ Existing evidence linking mechanical VTRFs to arterial thrombotic events is limited, with only some data on specific VTRFs (eg, surgery, trauma, immobilization) indicating a potential association. However, given the established contribution of traditional cardiovascular RFs to venous thromboembolism, it is plausible to hypothesize that the VTRFs may similarly play a role in arterial thrombosis.^11^

Perioperative stroke is an example of the latter relationship. Most cases result from well-established mechanisms, such as cardiac or aortic embolism, or hypoperfusion, particularly during high-risk cardiac or neurosurgical procedures.^27^ However, up to 13.9% of strokes following coronary artery bypass graft surgery are of an unknown cause, suggesting the existence of alternative mechanisms.^28^ Surgery can also precipitate cardiovascular events, as evidenced by myocardial injury after noncardiac surgery occurring in 17.9% of major surgeries.^29^ Both type I myocardial infarction caused by the rupture of atherosclerotic plaque and type 2 myocardial infarction caused by a mismatch between myocardial oxygen supply and demand due to the physiological stress of the perioperative period can occur.^30,31^ It can be hypothesized that the systemic stress response during surgery could trigger thromboinflammatory mechanisms eliciting acute arterial thrombosis in both the cardiovascular and cerebrovascular systems, potentially amplified by brain-heart axis interactions.

Trauma, particularly traumatic brain injury, is another well-established mechanical VTRF that can be associated with ischemic stroke, primarily through mechanisms such as arterial dissection, endothelial injury, vasospasm, thromboinflammation, and neuroinflammation.^32^ This association was found to be particularly prominent among patients under 50 years of age, which aligns with the findings of our study.^33^ Tissue injury due to trauma or surgery can initiate an inflammatory response that activates the coagulation cascade, affecting both arterial and venous systems.^34^ In addition, immobility, whether due to trauma, injury, or other causes, may impair the natural protective effects of physical activity against thrombotic events through multiple interconnected mechanisms.^35^ These include modulation of hemostatic processes, including viscosity, platelet function, regulation of coagulation factors, and systemic anti-inflammatory effects, which collectively safeguard against both arterial and VT.^36^ Mechanical VTRFs could, therefore, increase stroke risk by promoting systemic prothrombotic states that affect both vascular compartments.

While the risk of VT is highest in the immediate postoperative or posttraumatic period, it remains significantly elevated for an extended duration, even up to several weeks, although precise timing remains unidentified.^12,25,37^ Our findings indicate that the 14-day period preceding stroke is critical, whereas more distant periods do not correlate with an increased risk. This implies that the mechanism linking the mechanical trigger to the ischemic stroke could be more acute and transient than the well-described processes leading to VT. It is important to note, however, that the timing can vary between different VTRFs and individuals.

While paradoxical embolism via PFO originating from venous sources is commonly considered the primary mechanism for PFO-associated stroke, our findings suggest a more complex interplay between the RFs that precipitate VT and PFO. In adjusted analyses, recent mechanical VTRFs (≤14 days prestroke) were significantly associated with CIS in cases with clinically relevant PFO. Although the association did not reach statistical significance in participants without clinically relevant PFO, the consistent direction of effect across all models (adjusted OR point estimates, 1.29–1.59) suggests a potential trend toward elevated stroke risk in this subgroup as well. Furthermore, the absence of a significant interaction between PFO status and VTRFs implies that these factors may contribute additively, rather than synergistically, to stroke risk. This raises the possibility that VTRFs could elevate stroke risk through mechanisms independent of PFO status.

The presence of mechanical VTRFs within 14 days demonstrated a significant association with CIS in younger adults (18–39 years) after full adjustment, while no such association emerged in older cases. In addition, in exploratory analyses, we observed a significant interaction between mechanical VTRFs and traditional stroke RFs, with stronger associations in cases having ≤2 traditional RFs compared with those with >2. These observations likely reflect more atypical stroke mechanisms in younger patients with fewer traditional RFs. First, the well-established predominance of PFO-mediated stroke in younger individuals with limited traditional RFs suggests that our subgroups of patients aged 18 to 39 years and those with ≤2 cardiovascular RFs were likely enriched for PFO-positive cases.^13,38^ Second, the preserved vascular environment in these patients may allow venous thrombotic mechanisms to emerge as clinically significant contributors, while these pathways are typically masked by predominant atherosclerotic processes in higher-risk individuals.

In addition, we observed differences in the strength of the association between the sexes. In the unadjusted model, statistical significance was reached only in the male group, likely due to differences in statistical power and the divergent impact of individual mechanical VTRFs; for instance, immobilization was significant in men (*P*=0.018), while surgery was a highly significant trigger in women (*P*=0.002). However, after adjusting for female-specific factors, the point estimate of risk increased to its highest level (OR, 1.94 [95% CI, 0.81–4.63]), exceeding the effect size seen in the male subgroup. Although this did not reach statistical significance, it suggests that a biological signal likely exists but may be partially masked by competing sex-specific prothrombotic risks.

Inherited thrombophilias are well-established RFs for VT and exhibit synergistic risk when combined with acquired triggers (eg, surgery or pregnancy).^39–41^ Although this might suggest overrepresentation of thrombophilias in patients with CIS with VTRFs, existing evidence remains inconclusive.^14^ In our study, we found no significant differences in the baseline coagulation parameter levels among cases with and without mechanical VTRFs in any time interval within 90 days preceding stroke. This could suggest that mechanical VTRFs may independently contribute to stroke pathogenesis, despite an inherent prothrombotic predisposition. Notably, our thrombophilia samples were obtained at a median of 7 days poststroke, and thus, they may have missed hyperacute short-lasting abnormalities. In addition, antithrombotic therapies may have modified the coagulation profiles we were observing. The study’s focus on specific conventional coagulation markers also leaves open the possibility that VTRFs alter other aspects of hemostasis. Future investigations using holistic approaches such as thrombin generation assays could better characterize the interplay between mechanical triggers and the coagulation system.^42,43^

The findings of our study challenge the traditional view of distinctly different thrombosis mechanisms in arterial and venous systems, suggesting a more interconnected relationship. Some prior studies have observed a potential association between venous and arterial thrombotic events. A large 20-year Danish population–based cohort study demonstrated that both provoked and unprovoked venous thromboembolism significantly increased the risk of subsequent arterial cardiovascular events, including myocardial infarction and ischemic stroke, particularly during the first year following the thrombotic event.^44^ Another study similarly demonstrated associations between unprovoked venous thromboembolism and markers of atherosclerotic disease (asymptomatic carotid plaques and coronary artery calcification), suggesting that shared prothrombotic mechanisms may contribute to both arterial and venous thrombotic disorders.^45,46^ However, although our study demonstrates an association between mechanical VTRFs and CIS, it lacks direct evidence that these transient triggers precipitated VT before stroke onset. Further research is required to elucidate the precise pathophysiological mechanisms underlying the shared pathways between arterial and VT.

Our study demonstrates multiple methodological strengths. First, we analyzed a large, multicenter sample of young adults with radiologically confirmed first-ever CIS. All participants underwent standardized comprehensive assessments, including uniform diagnostic workup, structured data collection, and validated questionnaires. Furthermore, the extensive evaluation of both traditional and nontraditional RFs enabled robust adjustment for potential confounders in multivariable analyses, strengthening the validity of the findings. Nevertheless, there are several important limitations worth mentioning. First, the observational case-control design limits the ability to draw causal inferences in our study. Secondly, selection bias may affect both groups: cases through survivorship and the requirement to complete all diagnostic tests, and controls via a potential healthy-volunteer bias. Incomplete PFO status among controls further restricted PFO-stratified analyses to the screened subset. The prevalence of certain conditions may have been underestimated due to the reliance on retrospective self-reported data, which is susceptible to recall bias. For instance, mechanical VTRFs may be underreported by controls, while remote triggers may be reported inaccurately by both cases and controls. In addition, the study did not require systematic screening for deep VT in cases; therefore, we cannot determine whether the documented VTRFs had led to VT before stroke. Although our multicenter study is large for this specific population, more detailed subgroup analyses were limited by the restricted sample size and low frequency of certain event combinations. Given the exploratory nature of our subgroup and interaction analyses, the potential for type I errors due to multiple comparisons should be considered. The predominantly White European composition of our study population (94%) may limit its applicability to other young populations, particularly given the known racial/ethnic differences in both stroke RFs and outcomes. Finally, the absence of a standardized classification system for VTRFs hinders comparability across studies and the individual contribution of distinct exposures.

## Conclusions

Our study highlights the clinical importance of assessing mechanical VTRFs in stroke risk evaluation for young patients, particularly in those who are younger and lack conventional RFs. The 14-day period preceding ischemic stroke emerges as a window of heightened vulnerability, suggesting that acute mechanical triggers may initiate time-sensitive thrombotic pathways. While current clinical paradigms focus on mechanical VTRFs in the context of PFO-mediated paradoxical embolism, our findings raise the possibility that these transient triggers may contribute to stroke risk through mechanisms independent of PFO status. In addition, the occurrence of VTRFs was not associated with abnormal levels of prothrombotic RFs, such as coagulation markers. Future studies should prioritize elucidating the biological mechanisms linking mechanical VTRFs to arterial thrombosis. In parallel, clinical research should evaluate targeted prevention strategies during high-risk windows and whether only short-term antithrombotic therapy would be sufficient in young patients with CIS with temporal RFs and low burden of chronic traditional cardiovascular RFs.

## ARTICLE INFORMATION

### Acknowledgments

The authors are indebted to Anu Eräkanto for substantial technical help, study nurses, and all study participants for their volunteer participation in the study.

### Disclosures

Dr Tulkki reports grants from Finska Läkaresällskapet and Suomen Lääketieteen Säätiö. Dr Fonseca reports compensation from Bayer Healthcare for other services and travel support from Bayer Healthcare. Dr Huhtakangas reports grants from the Academy of Finland. Dr ten Cate reports compensation from the Synapse Research Institute for consultant services and stock holdings in CoagulationProfile. Dr Sinisalo reports compensation from Occlutech Holding AG for other services. The other authors report no conflicts.

### Supplemental Material

Tables S1–S12

Figures S1–S8

STROBE Checklist

## Supplementary Material



## Appendix

SECRETO Study Group (Principal Investigator [PI]): Vilnius University Tartu University Hospital, Tartu, Estonia: Külliki Karu, Janika Kõrv (PI), Liisa Kõrv, Piibe Muda, and Riina Vibo; Helsinki University Hospital, Helsinki, Finland: Shakar Kutal, Mika Lehto, Nicolas Martinez-Majander, Jani Pirinen, Jukka Putaala (PI), Janne Rapola, Tiina Sairanen, Maximillian Sihvo, Juha Sinisalo, Lauri Soinne, Satu Suihko, Marjaana Tiainen, Heli Tolppanen, Liisa Tomppo, Lauri Tulkki, and Suvi Tuohinen; Kuopio University Hospital and University of Eastern Finland, Kuopio, Finland: Jaana Autere, Marja Hedman, Nina Jaakonmäki, Pekka Jäkälä (PI), Tuuli Miettinen, and Ossi Nerg; Tampere University Hospital, Tampere, Finland: Heikki Numminen, Essi Ryödi, Tomi Sarkanen (PI), and Marko Virtanen; Turku University Hospital and University of Turku, Turku, Finland: Riitta Lautamäki, Antti Saraste, and Pauli Ylikotila (PI); Oulu University Hospital, Oulu, Finland: Jaana Huhtakangas, Juha Huhtakangas (PI), Ulla Junttola, and Laura Kytövuori; University Medical Greifswald, Greifswald, Germany: Raila Busch, Ulf Schminke, and Bettina von Sarnowski (PI); Attikon University Hospital, Athens, Greece: Alexandra Frogoudaki, Georgios Papadimitripoulos, and Georgios Tsivgoulis (PI); Azienda Unità Sanitaria Locale-IRCCS, Reggio Emilia, Italy: Teresa Grimaldi, Giovanni Malferrari, and Marialuisa Zedde (PI); University of Brescia, Brescia, Italy: Marina Colombi, CarloMario Lombardi, Alessandro Pezzini (PI), and Marco Ritelli; Vilnius University Hospital, Vilnius, Lithuania: Aleksandra Ekkert, Dalius Jatuzis, Rytis Masiliunas, Kristina Ryliskiene (PI), Julija Valančienė, and Diana Zakarkaite; Radboud University Medical Center, Nijmegen, the Netherlands: Frank-Erik de Leeuw (PI), Merel Ekker, Suzette Elias-Smale, and Myrna M.E. van Dongen; Haukeland University Hospital, Bergen, Norway: Annette Fromm, Eva Gerdts, Sahrai Saeed, and Ulrike Waje-Andreassen (PI); Hospital de Santa Maria, University of Lisbon, Lisbon, Portugal: Ana G. Almeida, Isabel Amorim, José Manuel Ferro, Ana Catarina Fonseca (PI), and André Paula; Torrecardenas University Hospital, Spain: Patricia Martínez-Sánchez (PI), Victoria Mejias Olmedo, Laura Amaya Pascasio, Raul Reyes Parrilla, and Elvira Carrión Rios; Sahlgrenska Academy at University of Gothenburg and Sahlgrenska University Hospital, Gothenburg, Sweden: Margareta Abrahamson, Odd Bech-Hanssen, Maria Davidson, Lukas Holmegaard, Mikael Jerndal, Katarina Jood, Annika Nordanstig, Petra Redfors (PI), and Turgut Tatlisumak; Istanbul University, Istanbul, Turkey: Esme Ekizoglu Turgut, Sezgin Mine, and Nilufer Yesilot (PI); Royal Stoke University Hospital, Stoke-on-Trent, United Kingdom: Phillip Ferdinand (PI), Cheryl Oxley, Zoltan Pencz, and Christine Roffe; Peterborough City Hospital, Peterborough, United Kingdom: Mohammad Hacque, Muhammad Khaled Hasan, Radim Licenik (PI), Peter Owusu-Agyei, and Santhosh Subramonian.
