# Supplementary material for: Association Between Recent Mechanical Risk Factors for Venous Thrombosis and Young-Onset Cryptogenic Ischemic Stroke
Source: Stroke. 2026 Aug 26;57(10):3010–23. doi: 10.1161/STROKEAHA.125.054299 (PMC13619135; doi:10.1161/STROKEAHA.125.054299)

SUPPLEMENTAL MATERIAL

Table S1. Control participants recruitment and patent foramen ovale (PFO) assessment by participating center.

Site name	City	Country	Controls screened for PFO / enrolled per center, N (%)
Tartu University Hospital	Tartu	Estonia	0/11 (0.0%)
Helsinki University Hospital	Helsinki	Finland	178/180 (98.9%)
Kuopio University Hospital	Kuopio	Finland	39/40 (97.5%)
Oulu University Hospital	Oulu	Finland	31/37 (83.8%)
Tampere University Hospital	Tampere	Finland	42/49 (85.7%)
Turku University Central Hospital	Turku	Finland	25/31 (80.6%)
University Medicine Greifswald	Greifswald	Germany	0/30 (0.0%)
Brescia Spedali Civili	Brescia	Italy	0/9 (0.0%)
Arcispedale Santa Maria Nuova IRCCS	Reggio Emilia	Italy	11/12 (91.7%)
Vilnius University Hospital Santaros Klinikos	Vilnius	Lithuania	0/10 (0.0%)
Haukeland Hospital	Bergen	Norway	25/25 (100.0%)
Hospital de Santa Maria, Centro Hospitalar Lisboa Norte	Lisbon	Portugal	0/17 (0.0%)

Torrecárdenas University Hospital	Almeria	Spain	12/12 (100.0%)
Sahlgrenska University Hospital	Gothenburg	Sweden	27/39 (69.2%)
Radboud University Medical Center	Nijmegen	The Netherlands	1/1 (100.0%)
Istanbul Faculty of Medicine, Istanbul University	Istanbul	Turkey	0/26 (0.0%)
Peterborough City Hospital	Peterborough	United Kingdom	1/6 (16.7%)
Royal Stoke University Hospital	Stoke-on-Trent	United Kingdom	1/11 (9.1%)

Table S2. Comparison of immobilization at different time periods prior to the index stroke between young cryptogenic ischemic stroke cases and stroke-free controls

**Different time points of immobilization before index stroke (2/3*)**	**All**			**Women**			**Men**		
	**Cases (n=546)**	**Controls (n=546)**	***P* value**	**Cases (n=258)**	**Controls (n=258)**	***P* value**	**Cases (n=288)**	**Controls (n=288)**	***P* value**
**Experienced within 14 days**	55 (10.1)	34 (6.3)	**0.021**	18 (7.0)	14 (5.4)	0.465	37 (12.9)	20 (7.0)	**0.018**
**Experienced within 15-30 days**	16 (2.9)	20 (3.7)	0.494	6 (2.3)	11 (4.3)	0.217	10 (3.5)	9 (3.1)	0.822
**Experienced within 31-60 days**	17 (3.1)	23 (4.2)	0.331	9 (3.5)	8 (3.1)	0.805	8 (2.8)	15 (5.2)	0.134
**Experienced within 61-90 days**	19 (3.5)	17 (3.1)	0.739	13 (5.1)	11 (4.3)	0.676	6 (2.1)	6 (2.1)	0.995

Data are n (%).

*Number of cases/controls with missing data if applicable.

Table S3. Comparison of injury at different time periods prior to the index stroke between young cryptogenic ischemic stroke cases and stroke-free controls

**Different time points of injury before index stroke (2/0*)**	**All**			**Women**			**Men**		
	**Cases (n=546)**	**Controls (n=546)**	***P* value**	**Cases (n=258)**	**Controls (n=258)**	***P* value**	**Cases (n=288)**	**Controls (n=288)**	***P* value**
**Experienced within 14 days**	12 (2.2)	4 (0.7)	**0.043**	3 (1.2)	1 (0.4)	0.315	9 (3.1)	3 (1.0)	0.078
**Experienced within 15-30 days**	10 (1.8)	6 (1.1)	0.310	5 (1.9)	3 (1.2)	0.476	5 (1.7)	3 (1.0)	0.470
**Experienced within 31-60 days**	15 (2.8)	3 (0.5)	**0.004**	7 (2.7)	1 (0.4)	**0.033**	8 (2.8)	2 (0.7)	0.054
**Experienced within 61-90 days**	12 (2.2)	14 (2.6)	0.698	6 (2.3)	8 (3.1)	0.588	6 (2.1)	6 (2.1)	0.990

Data are n (%).

*Number of cases/controls with missing data if applicable.

Table S4. Comparison of surgical treatment at different time periods prior to the index stroke between young cryptogenic ischemic stroke cases and stroke-free controls

**Different time points of surgical treatment before index stroke (1/1*)**	**All**			**Women**			**Men**		
	**Cases (n=546)**	**Controls (n=546)**	***P* value**	**Cases (n=258)**	**Controls (n=258)**	***P* value**	**Cases (n=288)**	**Controls (n=288)**	***P* value**
**Experienced within 14 days**	15 (2.8)	9 (1.7)	0.216	12 (4.7)	1 (0.4)	**0.002**	3 (1.0)	8 (2.8)	0.128
**Experienced within 15-30 days**	13 (2.4)	15 (2.8)	0.702	7 (2.7)	6 (2.3)	0.779	6 (2.1)	9 (3.1)	0.433
**Experienced within 31-60 days**	25 (4.6)	24 (4.4)	0.884	9 (3.5)	9 (3.5)	1.0	16 (5.6)	15 (5.2)	0.853
**Experienced within 61-90 days**	18 (3.3)	29 (5.3)	0.101	9 (3.5)	14 (5.4)	0.286	9 (3.1)	15 (5.2)	0.211

Data are n (%).

*Number of cases/controls with missing data if applicable.

Table S5. Comparison of non-surgical intervention at different time periods prior to the index stroke between young cryptogenic ischemic stroke cases and stroke-free controls

**Different time points of non-surgical intervention before index stroke (2/3*)**	**All**			**Women**			**Men**		
	**Cases (n=546)**	**Controls (n=546)**	***P* value**	**Cases (n=258)**	**Controls (n=258)**	***P* value**	**Cases (n=288)**	**Controls (n=288)**	***P* value**
**Experienced within 14 days**	10 (1.8)	13 (2.4)	0.524	8 (3.1)	11 (4.3)	0.472	2 (0.7)	2 (0.7)	0.997
**Experienced within 15-30 days**	8 (1.5)	13 (2.4)	0.269	3 (1.2)	9 (3.5)	0.077	5 (1.7)	4 (1.4)	0.733
**Experienced within 31-60 days**	15 (2.8)	17 (3.1)	0.716	9 (3.5)	9 (3.5)	0.987	6 (2.1)	8 (2.8)	0.593
**Experienced within 61-90 days**	16 (2.9)	14 (2.6)	0.715	8 (3.1)	7 (2.7)	0.805	8 (2.8)	7 (2.4)	0.788

Data are n (%).

*Number of cases/controls with missing data if applicable.

Table S6. Comparison of mechanical venous thrombosis risk factors (VTRFs) at different time points prior to the index stroke between young cryptogenic ischemic stroke cases and matched controls in different age groups

Different time points of any mechanical VTRFs before index stroke (No. of cases/controls with missing data if applicable)	Age 18-39 years			Age 40-49 years		
	**Cases (n=260)**	**Controls (n=217)**	***P* value**	**Cases (n=286)**	**Controls (n=329)**	***P* value**
**Any mechanical VTRFs (7/7)**						
**Experienced within 14 days**	40 (16.1)	21 (8.5)	**0.011**	43 (14.8)	31 (10.7)	0.155
**Experienced within 15-30 days**	19 (7.6)	19 (7.7)	1.000	17 (5.9)	24 (8.2)	0.311
**Experienced within 31-60 days**	23 (9.2)	21 (8.5)	0.878	23 (7.9)	26 (8.9)	0.761
**Experienced within 61-90 days**	18 (7.2)	24 (9.7)	0.337	25 (8.6)	22 (7.6)	0.761
**Immobilization (2/3)**						
**Experienced within 14 days**	22 (8.8)	16 (6.4)	0.405	33 (11.2)	18 (6.1)	**0.028**
**Experienced within 15-30 days**	10 (4.0)	11 (4.4)	1.000	6 (2.0)	9 (3.1)	0.581
**Experienced within 31-60 days**	6 (2.4)	9 (3.6)	0.581	11 (3.7)	14 (4.8)	0.839
**Experienced within 61-90 days**	12 (4.8)	11 (4.4)	1.000	7 (2.4)	6 (2.0)	1.000
**Injury (2/0)**						
**Experienced within 14 days**	9 (3.6)	1 (0.4)	**0.021**	3 (1.0)	3 (1.0)	1.000
**Experienced within 15-30 days**	4 (1.6)	3 (1.2)	0.687	6 (2.0)	3 (1.0)	0.508

**Experienced within 31-60 days**	8 (3.2)	3 (1.2)	0.227	7 (2.4)	0 (0.0)	**0.016**
**Experienced within 61-90 days**	9 (3.6)	10 (4.0)	1.000	3 (1.0)	4 (1.4)	1.000
**Surgical treatment (1/1)**						
**Experienced within 14 days**	6 (2.4)	4 (1.6)	0.754	9 (3.1)	5 (1.7)	0.424
**Experienced within 15-30 days**	8 (3.2)	3 (1.2)	0.227	5 (1.7)	12 (4.1)	0.143
**Experienced within 31-60 days**	15 (6.0)	9 (3.6)	0.307	10 (3.4)	15 (5.1)	0.424
**Experienced within 61-90 days**	8 (3.2)	14 (5.6)	0.263	10 (3.4)	15 (5.1)	0.405
**Non-surgical intervention (2/3)**						
**Experienced within 14 days**	9 (3.6)	3 (1.2)	0.146	1 (0.3)	10 (3.4)	**0.012**
**Experienced within 15-30 days**	4 (1.6)	6 (2.4)	0.754	4 (1.4)	7 (2.4)	0.549
**Experienced within 31-60 days**	5 (2.0)	8 (3.2)	0.581	10 (3.4)	9 (3.1)	1.000
**Experienced within 61-90 days**	6 (2.4)	6 (2.4)	1.000	10 (3.4)	8 (2.7)	0.815

Data are n (%).

Table S7. Odds ratios (ORs) and 95% confidence intervals (CIs) from conditional logistic regression on the association between any mechanical venous thrombosis risk factors (VTRFs) (excluding the category of non-surgical interventions) in different time periods and cryptogenic ischemic stroke in the entire sample. Sensitivity analysis

	**Model 1: unadjusted**		**Model 2: adjusted for sex, age and level of education**		**Model 3: adjusted for sex, age, level of education, and traditional stroke risk factors***		**Model 4: adjusted for sex, age, level of education, traditional* and non-traditional† stroke risk factors**	
	**OR (95% CI)**	***P* value**	**OR (95% CI)**	***P* value**	**OR (95% CI)**	***P* value**	**OR (95% CI)**	***P* value**
**Any mechanical VTRFs within 14 days**	1.89 (1.24-2.88)	**0.003**	1.83 (1.17-2.87)	**0.008**	1.76 (1.07-2.87)	**0.025**	1.83 (1.07-3.11)	**0.026**
**Any mechanical VTRFs within 15-90 days**	0.92 (0.67-1.27)	0.619	0.91 (0.65-1.28)	0.600	0.81 (0.56-1.18)	0.277	0.69 (0.46-1.04)	0.078

*Traditional stroke risk factors included in the analysis were abdominal obesity, current tobacco smoking, depression, heavy alcohol consumption, hypertension, physical inactivity, psychosocial stress, unhealthy diet.

†Non-traditional stroke risk factors included in the analysis were chronic multi-organ disorder (any of inflammatory bowel disease, other autoimmune, chronic kidney/liver disease, or hematologic disease/thrombophilia), history of venous thromboembolism, illicit drug use, malignancy and migraine with aura.

Table S8. Odds ratios (ORs) and 95% confidence intervals (CIs) from conditional logistic regression on the association between mechanical venous thrombosis risk factors (VTRFs) in different time points and cryptogenic ischemic stroke in men

	**Model 1: unadjusted**		**Model 2: adjusted for sex, age and level of education**		**Model 3: adjusted for age, level of education, and *traditional stroke risk factors**		**Model 4: adjusted for age, level of education, *traditional and †non-traditional stroke risk factors**	
	**OR (95% CI)**	***P* value**	**OR (95% CI)**	***P* value**	**OR (95% CI)**	***P* value**	**OR (95% CI)**	***P* value**
**Any mechanical VTRFs within 14 days**	1.77 (1.04-3.04)	**0.037**	1.62 (0.92-2.84)	0.093	1.69 (0.88-3.22)	0.113	1.71 (0.85-3.45)	0.136
**Any mechanical VTRFs within 15-90 days**	0.93 (0.60-1.42)	0.727	0.96 (0.61-1.52)	0.870	0.79 (0.47-1.35)	0.388	0.77 (0.43-1.36)	0.365

*Traditional stroke risk factors included in the analysis were abdominal obesity, current tobacco smoking, depression, heavy alcohol consumption, hypertension, physical inactivity, psychosocial stress, unhealthy diet.

†Non-traditional stroke risk factors included in the analysis were chronic multi-organ disorder (any of inflammatory bowel disease, other autoimmune, chronic kidney/liver disease, or hematologic disease/thrombophilia), history of venous thromboembolism, illicit drug use, malignancy and migraine with aura.

Table S9. Odds ratios (ORs) and 95% confidence intervals (CIs) from conditional logistic regression on the association between mechanical venous thrombosis risk factors (VTRFs) in different time points and cryptogenic ischemic stroke in women

	**Model 1: unadjusted**		**Model 2: adjusted for sex, age and level of education**		**Model 3: adjusted for age, level of education, and *traditional stroke risk factors**		**Model 4: adjusted for age, level of education, *traditional and †non-traditional stroke risk factors**		**Model 5: adjusted for age, level of education, *traditional, †non-traditional stroke risk factors and ‡female-sex-specific risk factors**	
	**OR (95% CI)**	***P* value**	**OR (95% CI)**	***P* value**	**OR (95% CI)**	***P* value**	**OR (95% CI)**	***P* value**	**OR (95% CI)**	***P* value**
**Any mechanical VTRFs within 14 days**	1.79 (0.97-3.29)	0.062	1.82 (0.95-3.47)	0.069	1.78 (0.87-3.63)	0.114	1.70 (0.75-3.89)	0.205	1.94 (0.81-4.63)	0.135
**Any mechanical VTRFs within 15-90 days**	1.09 (0.71-1.68)	0.694	1.03 (0.65-1.63)	0.898	1.01 (0.61-1.69)	0.963	0.89 (0.48-1.66)	0.710	0.90 (0.46-1.78	0.766

*Traditional stroke risk factors included in the analysis were abdominal obesity, current tobacco smoking, depression, heavy alcohol consumption, hypertension, physical inactivity, psychosocial stress, unhealthy diet.

†Non-traditional stroke risk factors included in the analysis were chronic multi-organ disorder (any of inflammatory bowel disease, other autoimmune, chronic kidney/liver disease, or hematologic disease/thrombophilia), history of venous thromboembolism, illicit drug use, malignancy and migraine with aura.

[‡]Female-sex specific risk factors included in the analysis were any perinatal condition (pregnancy diabetes, hypertension, pre-eclampsia, eclampsia, or HELLP (Hemolysis, Elevated Liver enzymes and Low Platelets) syndrome) or any prestroke estrogen preparate use.

Table S10. Odds ratios (ORs) and 95% confidence intervals (CIs) from conditional logistic regression on the association between mechanical venous thrombosis risk factors (VTRFs) in different time points and cryptogenic ischemic stroke in different age groups

	**Model 1: unadjusted**		**Model 2: adjusted for sex, age and level of education**		**Model 3: adjusted for age, level of education, and *traditional stroke risk factors**		**Model 4: adjusted for age, level of education, *traditional and †non-traditional stroke risk factors**	
	**OR (95% CI)**	***P* value**	**OR (95% CI)**	***P* value**	**OR (95% CI)**	***P* value**	**OR (95% CI)**	***P* value**
**Patients 18-39 years of age**								
**Any mechanical VTRFs within 14 days**	2.24 (1.21-4.12)	**0.010**	2.14 (1.10-4.16)	**0.025**	2.23 (1.03-4.80)	**0.041**	2.36 (1.02-5.46)	**0.046**
**Any mechanical VTRFs within 15-90 days**	1.07 (0.67-1.69)	0.784	1.07 (0.65-1.77)	0.779	0.85 (0.48-1.51)	0.583	0.85 (0.44-1.63)	0.625
**Patients 40-49 years of age**								
**Any mechanical VTRFs within 14 days**	1.47 (0.86-2.53)	0.162	1.57 (0.89-2.78)	0.121	1.49 (0.80-2.81)	0.212	1.50 (0.73-3.08)	0.271
**Any mechanical VTRFs within 15-90 days**	0.95 (0.64-1.43)	0.819	0.95 (0.62-1.45)	0.804	0.92 (0.57-1.49)	0.723	0.86 (0.49-1.50)	0.585

*Traditional stroke risk factors included in the analysis were abdominal obesity, current tobacco smoking, depression, heavy alcohol consumption, hypertension, physical inactivity, psychosocial stress, unhealthy diet.

[†]Non-traditional stroke risk factors included in the analysis were chronic multi-organ disorder (any of inflammatory bowel disease, other autoimmune, chronic kidney/liver disease, or hematologic disease/thrombophilia), history of venous thromboembolism, illicit drug use, malignancy and migraine with aura.

Table S11. Exploratory subgroup analyses of the association between any mechanical venous thrombosis risk factors (VTRFs) within 14 days preceding stroke and cryptogenic ischemic stroke

**Subgroup/Risk factor**	**Mechanical VTRFs within 14 days**<br><br>**Number of patients/controls**	**Unadjusted OR (95% CI)**	***P* for interaction***
**Age groups**			0.254
18-39 years	40/21	1.48 (0.82-2.66)	
40-49 years	43/31	1.89 (1.16-3.11)	
**Sex**			0.993
Female	36/24	1.59 (0.91-2.78)	
Male	47/28	1.79 (1.07-2.98)	
**Clinically relevant PFO**			0.322
Yes	40/11	0.76 (0.35-1.65)	
No	38/33	1.46 (0.88-2.43)	
**Low burden of traditional stroke risk factors**			**0.044**
Yes	37/22	2.97 (1.67-5.26)	
No	44/26	0.96 (0.56-1.65)	
**Low burden of non-traditional stroke risk factors**			0.672
Yes	67/49	1.61 (1.08-2.41)	
No	16/3	1.70 (0.43-6.69)	

CI, confidence interval; OR, odds ratio; PFO, patent foramen ovale

**P* value from logistic regression model adjusted for age, sex, level of education, traditional and non-traditional stroke risk factors.

Table S12. Abnormalities in baseline coagulation parameters in young cryptogenic ischemic stroke patients with and without mechanical venous thrombosis risk factors (VTRFs)

Abnormalities in baseline coagulation parameters (No. of cases with missing data if applicable)	No mechanical VTRFs within 90 days	Any mechanical VTRFs within 14 days	Any mechanical VTRFs within 15-90 days	*P* value
**Antithrombin III deficiency (94)**	7 (2.2)	3 (3.9)	5 (4.2)	0.471
**Protein C deficiency (86)**	9 (2.8)	0	1 (0.8)	0.165
**Protein S deficiency (91)**	10 (3.1)	3 (3.8)	5 (4.2)	0.855
**Elevated levels of factor VIII (325)**	40 (24.1)	15 (35.7)	21 (28.4)	0.301
**Factor II G20210A mutation (112)**				
Heterozygous	3 (1.0)	1 (1.4)	1 (0.9)	0.947
Homozygous	0	0	0	
**Factor V G1691A mutation (109)**				
Heterozygous	14 (4.5)	0	3 (2.6)	0.119
Homozygous	0	0	1 (0.9)	
**Hyperhomocysteinemia (179)**	0	0	0	NA
**Any of antiphospholipid antibodies* (19)**	25 (6.6)	9 (10.8)	10 (7.9)	0.402

NA, not applicable.

Data are n (%).

*Lupus anticoagulant (LA) or anticardiolipin antibody (aCL) or anti-β2-glycoprotein-I (anti-β2GPI).

Figure S1. Study flow diagram of participant selection and site-specific enrollment

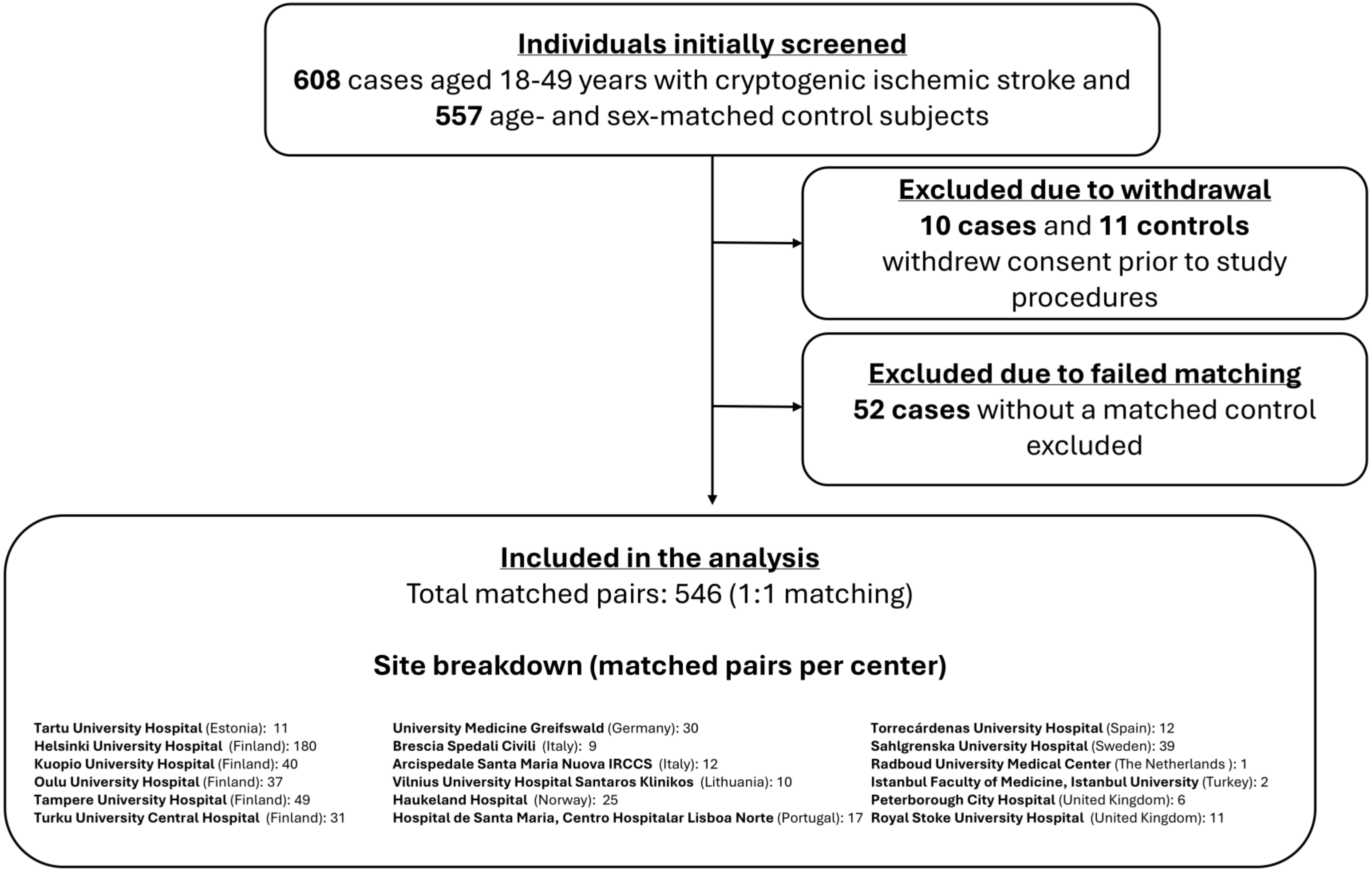

Figure S2. Distribution of baseline coagulation markers in young cryptogenic ischemic stroke patients with and without mechanical venous thrombosis risk factors (VTRFs), with those with VTRFs dichotomized by their temporal interval from stroke (within 14 days vs. 15-90 days)

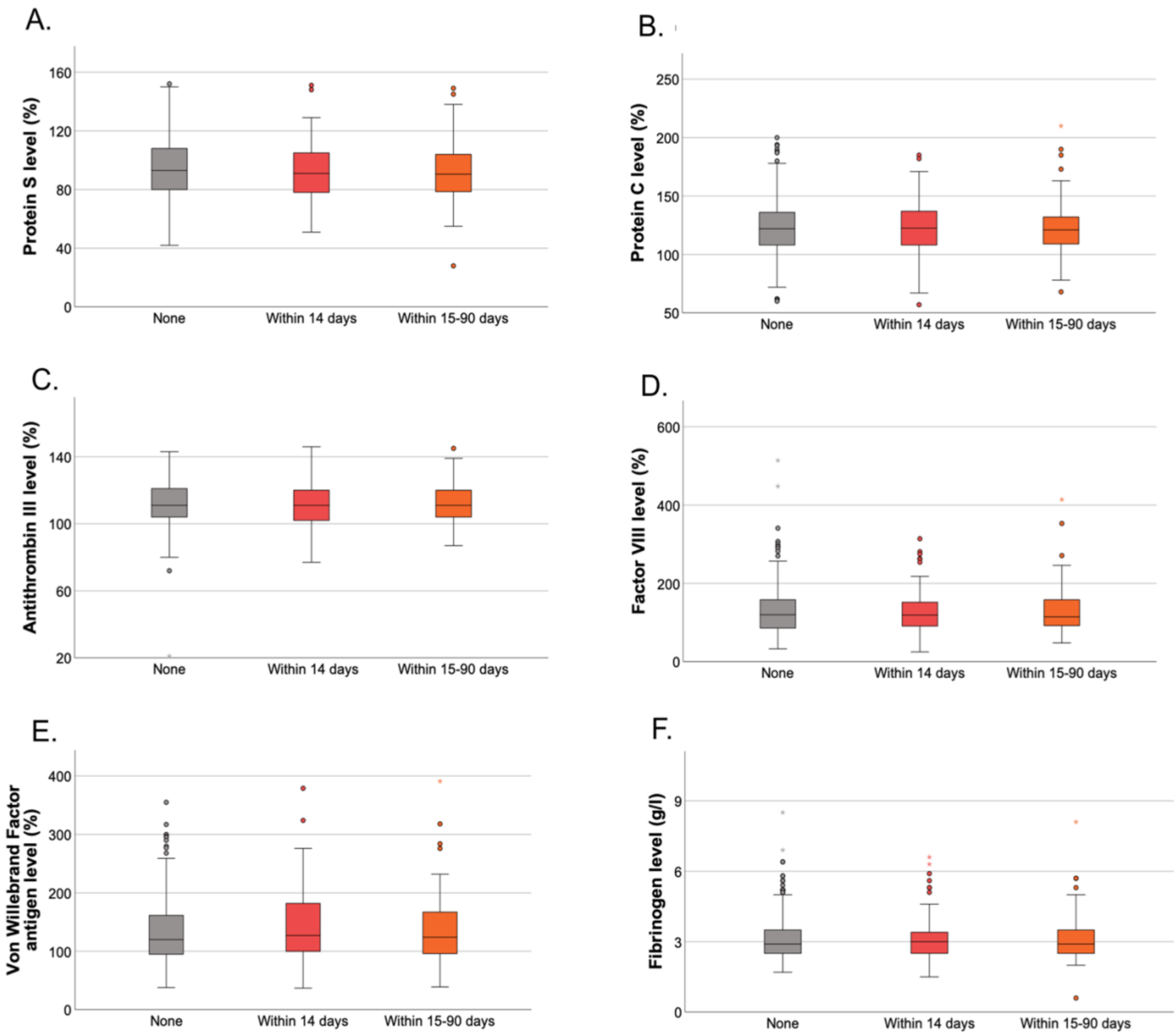

Figure S3. Distribution of baseline coagulation markers in a subgroup of cryptogenic ischemic stroke patients aged 18–39 years with and without mechanical venous thrombosis risk factors (VTRFs), with those with VTRFs dichotomized by their temporal interval from stroke (within 14 days vs. 15-90 days)

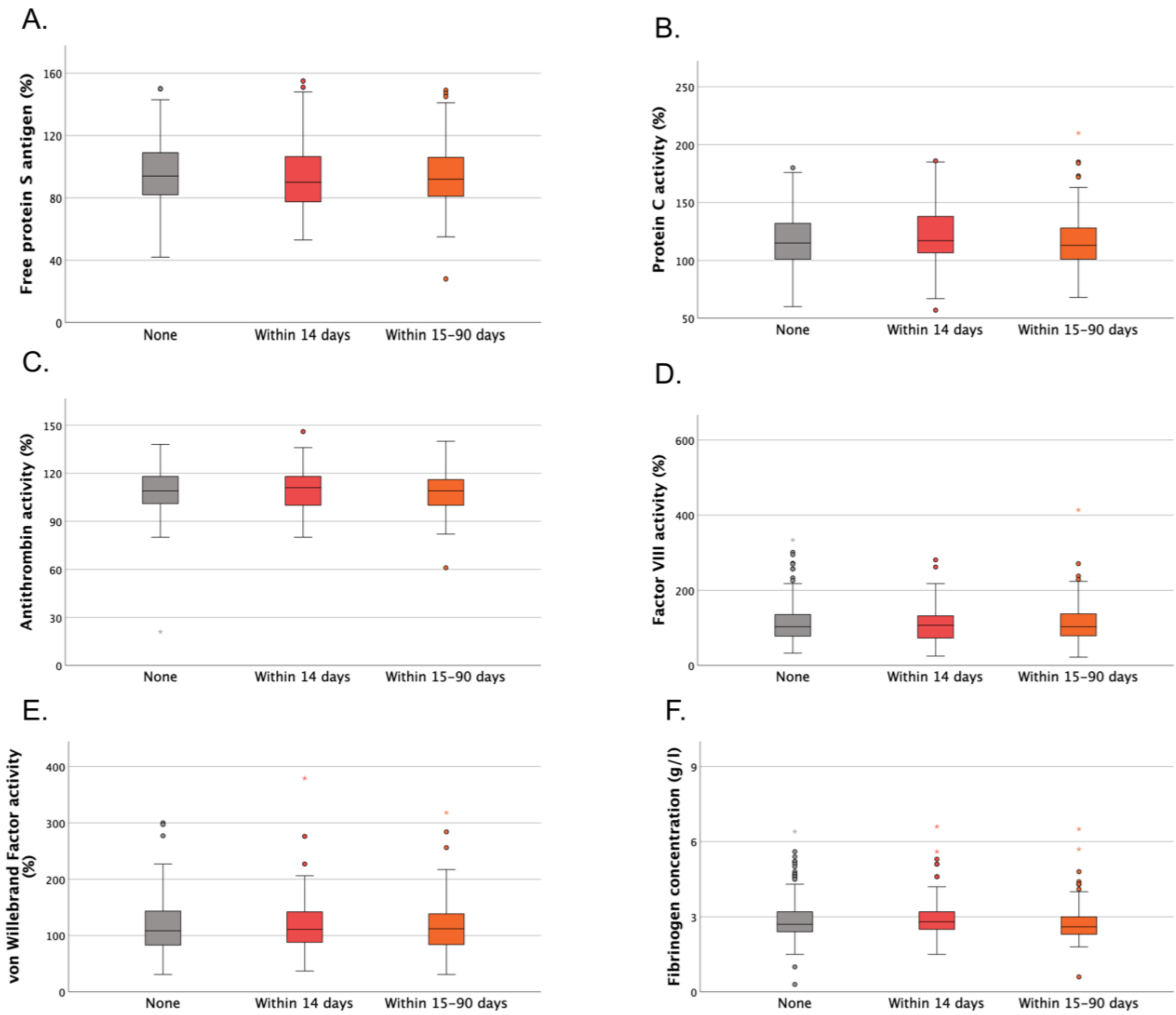

Figure S4. Distribution of baseline coagulation markers in a subgroup of cryptogenic ischemic stroke patients aged 40-49 years with and without mechanical venous thrombosis risk factors (VTRFs), with those with VTRFs dichotomized by their temporal interval from stroke (within 14 days vs. 15-90 days)

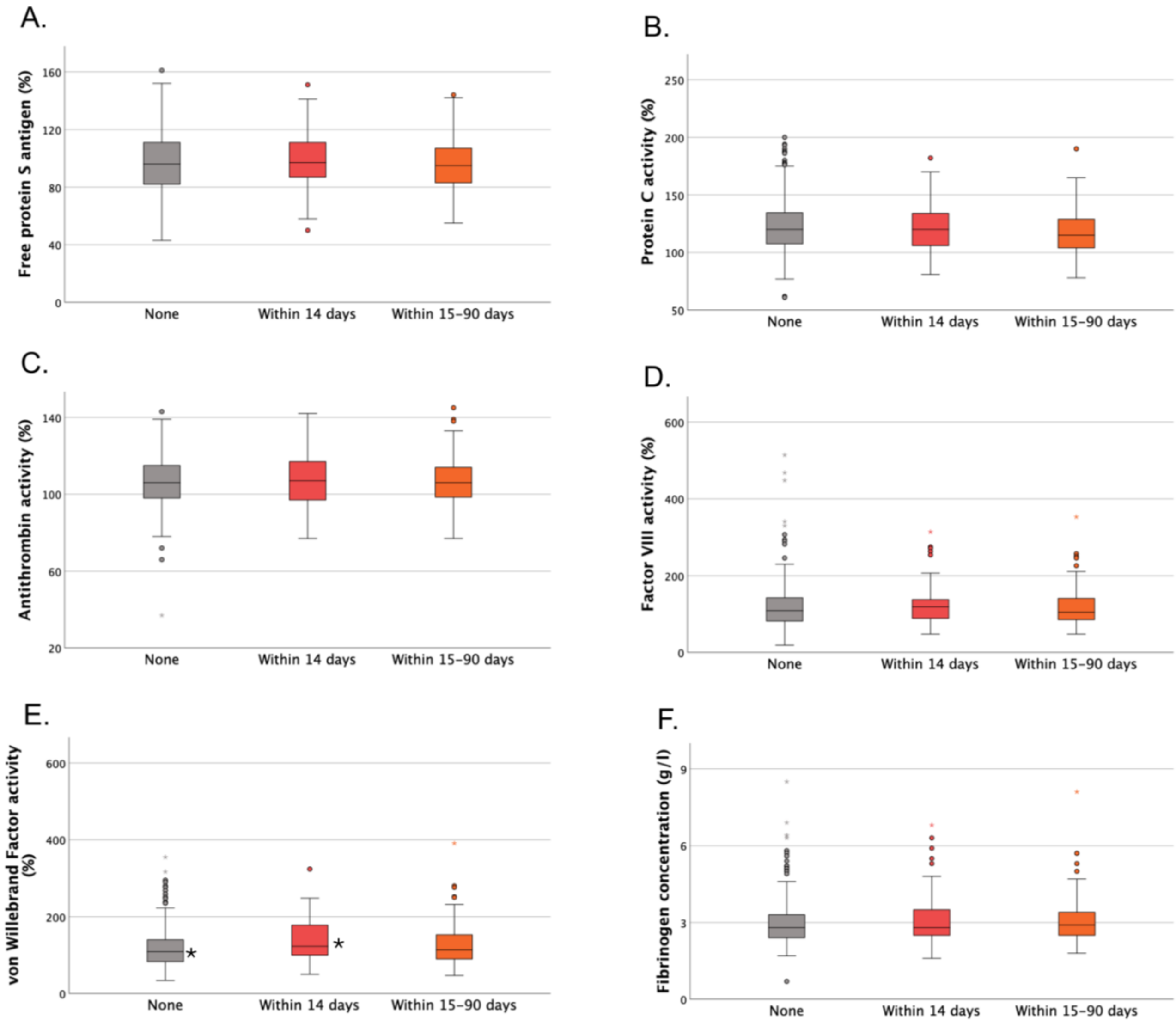

*Statistically significant difference between groups (*P*<0.05).

Figure S5. Distribution of baseline coagulation markers in a subgroup of young cryptogenic ischemic stroke patients with a high burden of traditional stroke risk factors, with and without mechanical venous thrombosis risk factors (VTRFs), with those with VTRFs dichotomized by their temporal interval from stroke (within 14 days vs. 15-90 days)

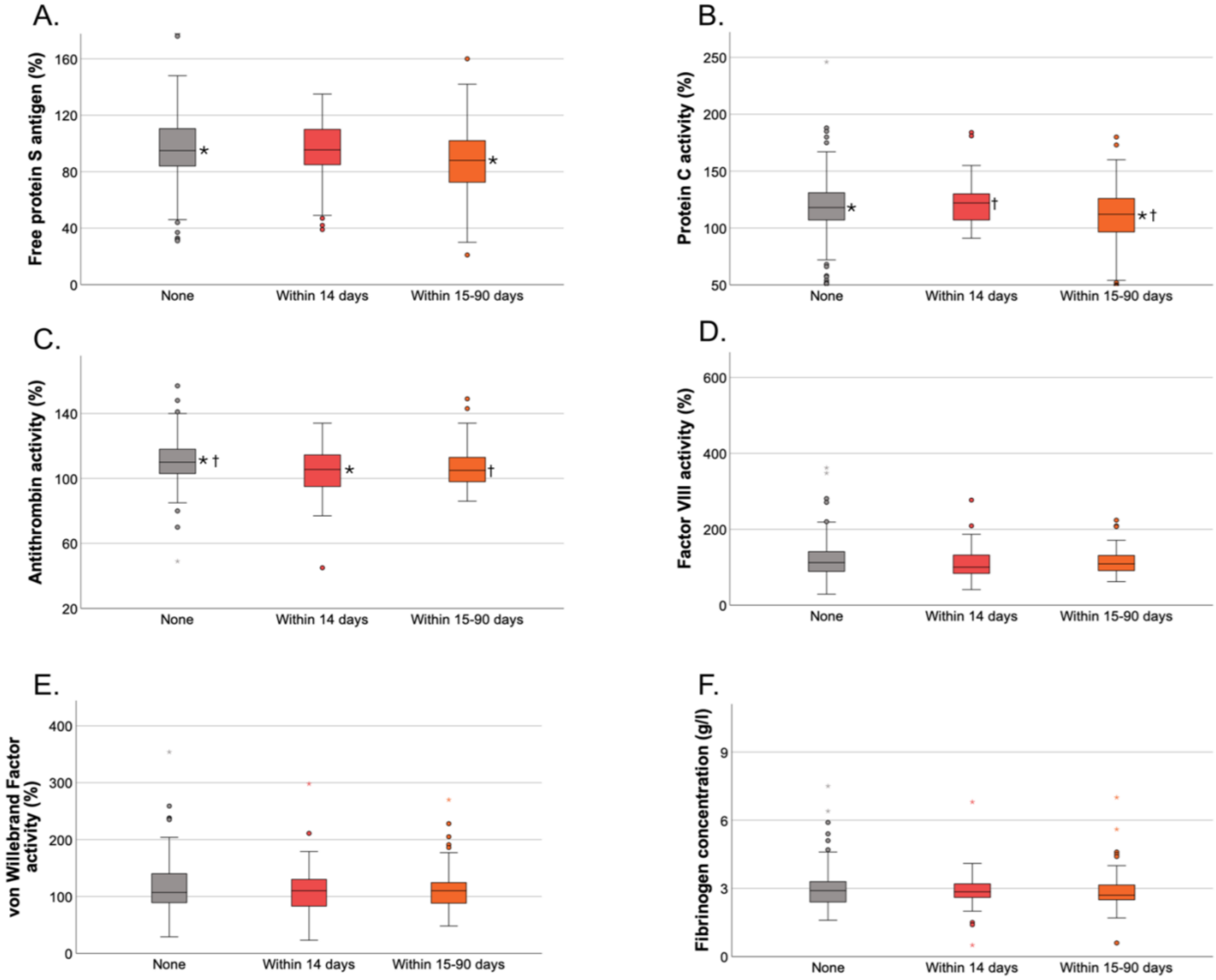

*,†Statistically significant difference between groups (*P*<0.05).

Figure S6. Distribution of baseline coagulation markers in a subgroup of young cryptogenic ischemic stroke patients with a low burden of traditional stroke risk factors, with and without mechanical venous thrombosis risk factors (VTRFs), with those with VTRFs dichotomized by their temporal interval from stroke (within 14 days vs. 15-90 days)

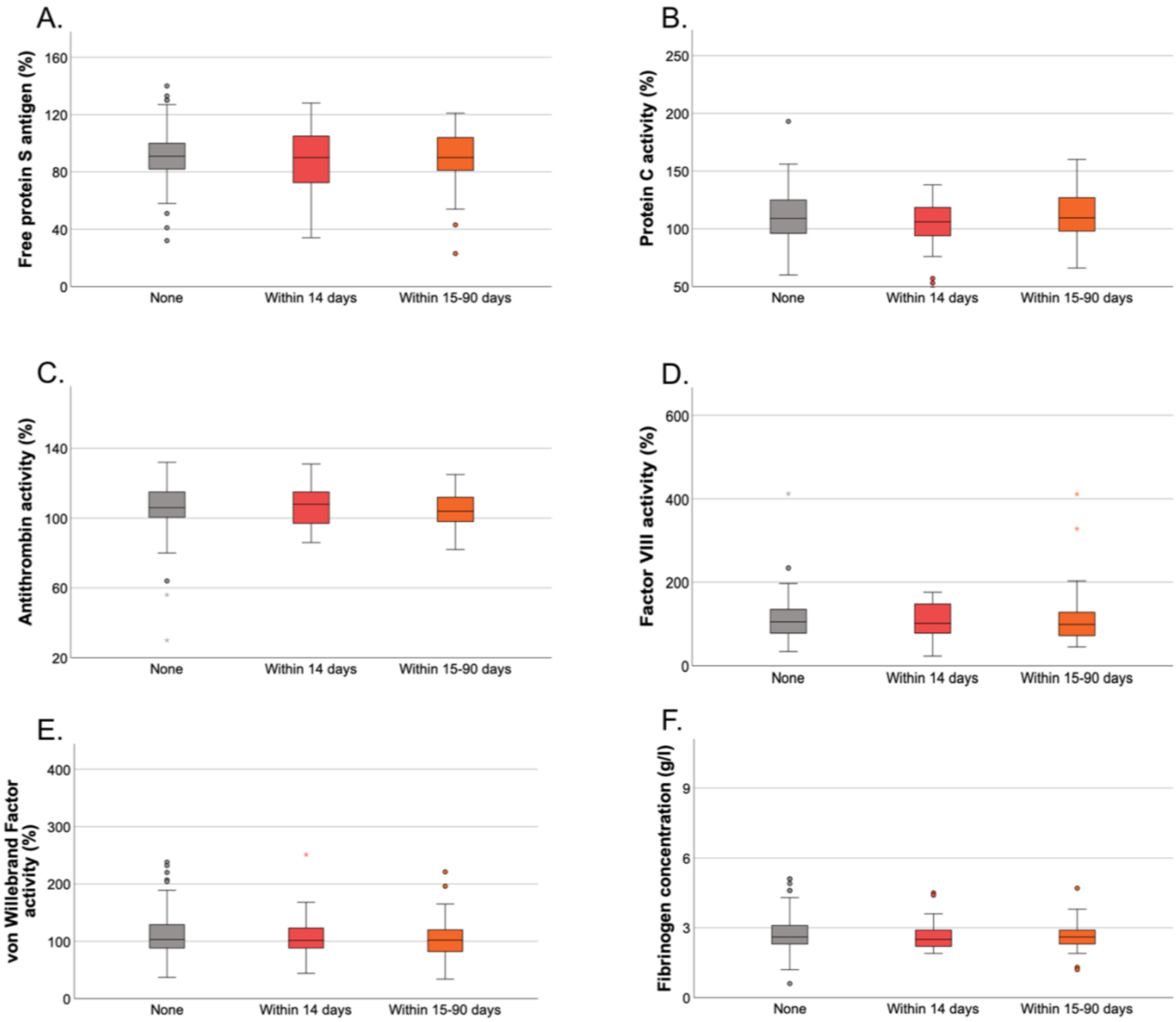

Figure S7. Distribution of baseline coagulation markers in a subgroup of young cryptogenic ischemic stroke patients who do not have a clinically relevant patent foramen ovale, with and without mechanical venous thrombosis risk factors (VTRFs), with those with VTRFs dichotomized by their temporal interval from stroke (within 14 days vs. 15-90 days)

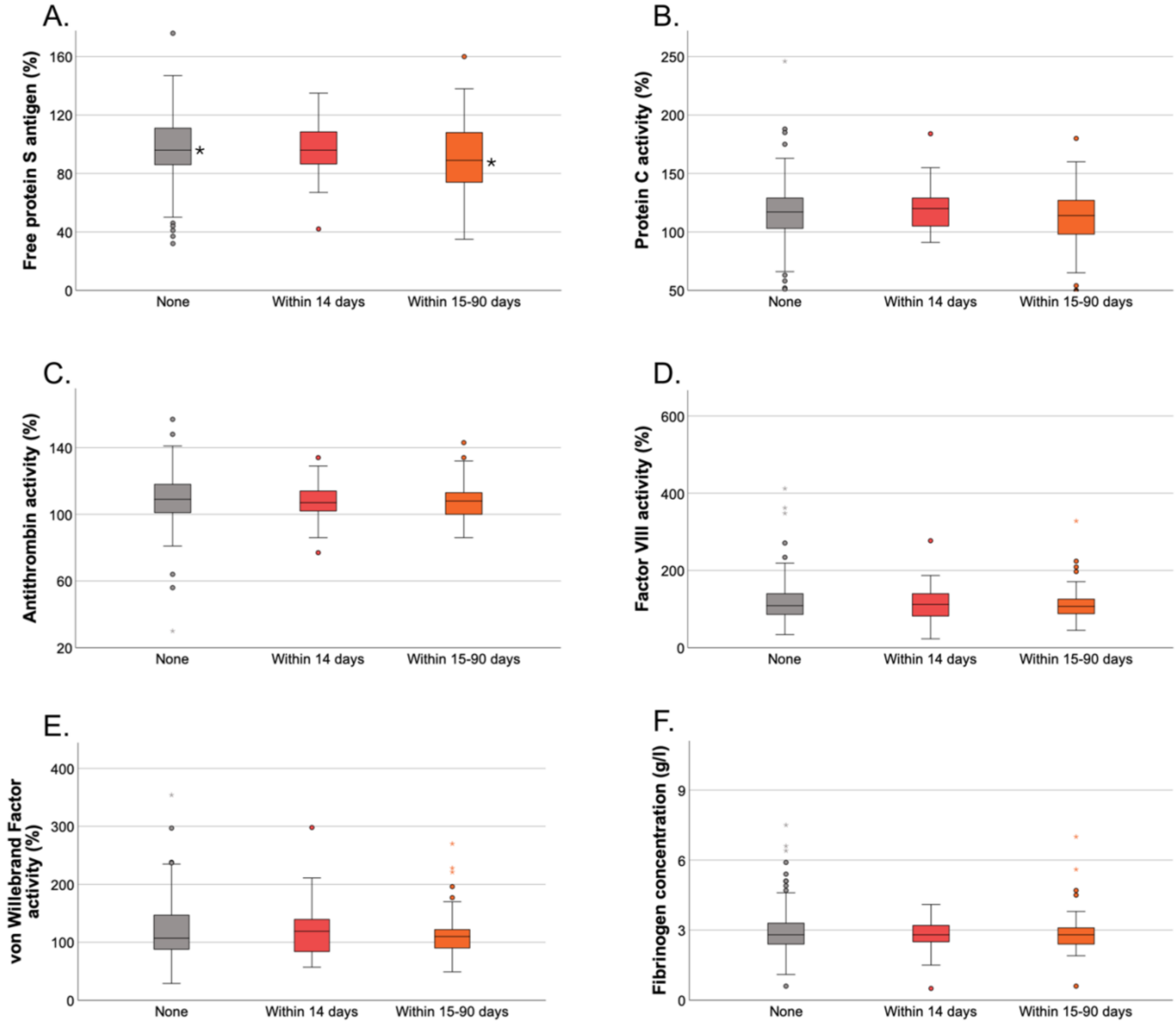

*Statistically significant difference between groups (*P*<0.05).

Figure S8. Distribution of baseline coagulation markers in a subgroup of young cryptogenic ischemic stroke patients who have a clinically relevant patent foramen ovale, with and without mechanical venous thrombosis risk factors (VTRFs), with those with VTRFs dichotomized by their temporal interval from stroke (within 14 days vs. 15-90 days)

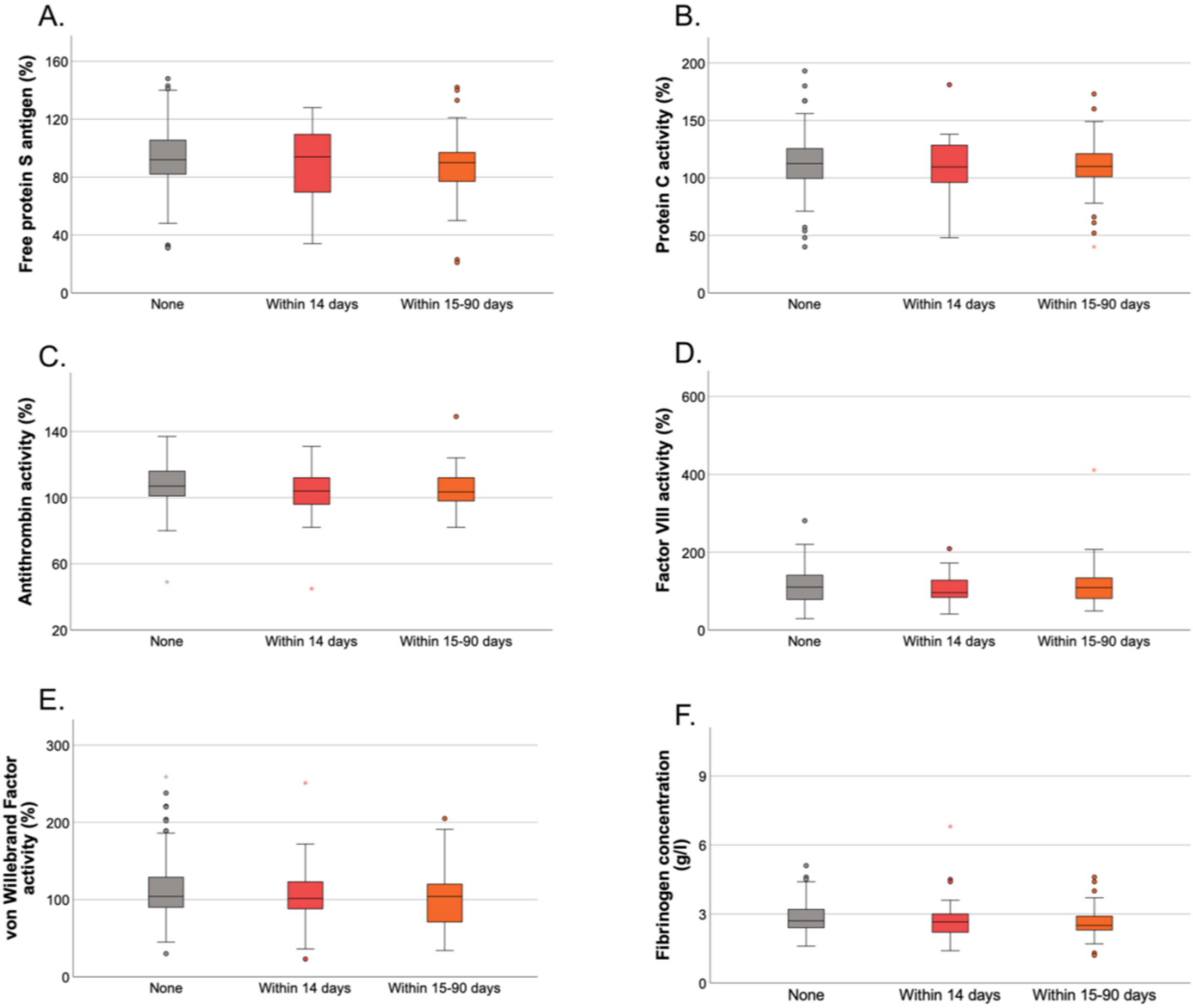

Supplement: Supplementary file 1 [file str-57-3010-s001.pdf]
