# Supplementary material for: Association Between Recent Mechanical Risk Factors for Venous Thrombosis and Young-Onset Cryptogenic Ischemic Stroke
Source: Stroke. 2026 Aug 26;57(10):3010–23. doi: 10.1161/STROKEAHA.125.054299 (PMC13619135; doi:10.1161/STROKEAHA.125.054299)
Supplement: Supplementary file 2 [file str-57-3010-s002.pdf]

STROBE Statement—checklist of items that should be included in reports of observational studies

	Item No.	Recommendation	Page No., lines, comments
**Title and abstract**	1	(*a*) Indicate the study's design with a commonly used term in the title or the abstract	Page No. 4; Line 67.
		(*b*) Provide in the abstract an informative and balanced summary of what was done and what was found	Page No. 5; Lines 85-87.
**Introduction**			
Background/rationale	2	Explain the scientific background and rationale for the investigation being reported	Page No. 5, 6; Lines 96-99, 121-122.
Objectives	3	State specific objectives, including any prespecified hypotheses	Page No. 6; Lines 122-126.
**Methods**			
Study design	4	Present key elements of study design early in the paper	Page No. 7; Lines 134-143.
Setting	5	Describe the setting, locations, and relevant dates, including periods of recruitment, exposure, follow-up, and data collection	Page No. 7; Lines 136-139.
Participants	6	(*a*) *Cohort study*—Give the eligibility criteria, and the sources and methods of selection of participants. Describe methods of follow-up<br>*Case-control study*—Give the eligibility criteria, and the sources and methods of case ascertainment and control selection. Give the rationale for the choice of cases and controls<br>*Cross-sectional study*—Give the eligibility criteria, and the sources and methods of selection of participants	Page No. 7, 8; Lines 136-137, 139-143, 151-152.
		(*b*) *Cohort study*—For matched studies, give matching criteria and number of exposed and unexposed<br>*Case-control study*—For matched studies, give matching criteria and the number of controls per case	Page No. 7; Lines 138-140.
Variables	7	Clearly define all outcomes, exposures, predictors, potential confounders, and effect modifiers. Give diagnostic criteria, if applicable	Page No. 7-11; Lines 145-150, 161-165, 186-188, 198-200, 204-210, 215-218.

Data sources/ measurement	8	For each variable of interest, give sources of data and details of methods of assessment (measurement). Describe comparability of assessment methods if there is more than one group	Page No. 10, 11; Lines 198-200, 204-210, 215-218. *The precise diagnostic criteria for each assessed risk factor are listed in the "Methods" section on pages 8-10.*
Bias	9	Describe any efforts to address potential sources of bias	Page No. 7, 8, 10, 11; Lines 151-152, 161-164, 210-213, 218-221.
Study size	10	Explain how the study size was arrived at	Page No. 7; Lines 134-138.

Continued on next page

Quantitative variables	11	Explain how quantitative variables were handled in the analyses. If applicable, describe which groupings were chosen and why	Page No. 11-13; Lines 231-234, 239-243, 251-253, 259-261, 264, 268-270, 275-277.
Statistical methods	12	(*a*) Describe all statistical methods, including those used to control for confounding	Page No. 11-13; Lines 229-239, 244-246, 251-261, 264, 266-268, 270-273, 275-277.
		(*b*) Describe any methods used to examine subgroups and interactions	Page No. 11-13; Lines 231, 233-234, 251-257, 259-261, 263-264, 266-268, 270-273, 275-277.
		(*c*) Explain how missing data were addressed	Page No. 11; Lines 235-236.
		(*d*) *Cohort study*—If applicable, explain how loss to follow-up was addressed<br>*Case-control study*—If applicable, explain how matching of cases and controls was addressed<br>*Cross-sectional study*—If applicable, describe analytical methods taking account of sampling strategy	Page No. 7; Lines 139-140.
		(*e*) Describe any sensitivity analyses	Page No. 12; Lines 259-262.
**Results**			
Participants	13	(a) Report numbers of individuals at each stage of study—eg numbers potentially eligible, examined for eligibility, confirmed eligible, included in the study, completing follow-up, and analysed	Page No.14; Lines 279-282.
		(b) Give reasons for non-participation at each stage	*Figure S1 ("Supplemental Material")*
		(c) Consider use of a flow diagram	*Figure S1 ("Supplemental Material")*
Descriptive data	14	(a) Give characteristics of study participants (eg demographic, clinical, social) and information on exposures and potential confounders	Page No. 14, 15, 17, 18; Lines 284-312, 357-367.
		(b) Indicate number of participants with missing data for each variable of interest	Page No. 32-35.<br>*Table 1, Table 2.*

		(c) *Cohort study*—Summarise follow-up time (eg, average and total amount)	NA
Outcome data	15	*Cohort study*—Report numbers of outcome events or summary measures over time	NA
		Case-control study—Report numbers in each exposure category, or summary measures of exposure	Page No. 34-35. *Table 2.*
		Cross-sectional study—Report numbers of outcome events or summary measures	NA
Main results	16	(*a*) Give unadjusted estimates and, if applicable, confounder-adjusted estimates and their precision (eg, 95% confidence interval). Make clear which confounders were adjusted for and why they were included	Page No. 15,16; Lines 314-322.
		(*b*) Report category boundaries when continuous variables were categorized	Page No. 12; Lines 239-243, 251-252.
		(*c*) If relevant, consider translating estimates of relative risk into absolute risk for a meaningful time period	NA

Continued on next page

Other analyses	17	Report other analyses done—eg analyses of subgroups and interactions, and sensitivity analyses	Page No. 14-18; Lines 290-297, 300-312, 320-334, 336-338, 340-347, 349-354, 363-367.
**Discussion**			
Key results	18	Summarise key results with reference to study objectives	Page No. 19; Lines 371-377, 379-381.
Limitations	19	Discuss limitations of the study, taking into account sources of potential bias or imprecision. Discuss both direction and magnitude of any potential bias	Page No. 24; Lines 486-503.
Interpretation	20	Give a cautious overall interpretation of results considering objectives, limitations, multiplicity of analyses, results from similar studies, and other relevant evidence	Please see the "Discussion" section on pages 19-24.
Generalisability	21	Discuss the generalisability (external validity) of the study results	Page No. 23-25; Lines 464-468, 478-480, 505-507, 513-517.
**Other information**			
Funding	22	Give the source of funding and the role of the funders for the present study and, if applicable, for the original study on which the present article is based	Page No. 30; Lines 553-555.
